# Regulation of CTLs/Tregs via Highly Stable and Ultrasound‐Responsive Cerasomal Nano‐Modulators for Enhanced Colorectal Cancer Immunotherapy

**DOI:** 10.1002/advs.202400485

**Published:** 2024-03-29

**Authors:** Jinxia Zhang, Lihong Sun, Ling Jiang, Xinxin Xie, Yuan Wang, Ruiqi Wu, Qingshuang Tang, Suhui Sun, Shiwei Zhu, Xiaolong Liang, Ligang Cui

**Affiliations:** ^1^ Institute of Medical Technology Peking University Health Science Center Beijing 100010 P. R. China; ^2^ Department of Ultrasound Peking University Third Hospital Beijing 100191 P. R. China

**Keywords:** cerasomes, immunoregulation, immunotherapy, sonodynamic therapy, ultrasound‐responsive

## Abstract

Immunotherapy is showing good potential for colorectal cancer therapy, however, low responsive rates and severe immune‐related drug side effects still hamper its therapeutic effectiveness. Herein, a highly stable cerasomal nano‐modulator (DMC@P‐Cs) with ultrasound (US)‐controlled drug delivery capability for selective sonodynamic‐immunotherapy is fabricated. DMC@P‐Cs’ lipid bilayer is self‐assembled from cerasome‐forming lipid (CFL), pyrophaeophorbid conjugated lipid (PL), and phospholipids containing unsaturated chemical bonds (DOPC), resulting in US‐responsive lipid shell. Demethylcantharidin (DMC) as an immunotherapy adjuvant is loaded in the hydrophilic core of DMC@P‐Cs. With US irradiation, reactive oxygen species (ROS) can be effectively generated from DMC@P‐Cs, which can not only kill tumor cells for inducing immunogenic cell death (ICD), but also oxidize unsaturated phospholipids‐DOPC to change the permeability of the lipid bilayers and facilitate controlled release of DMC, thus resulting in down‐regulation of regulatory T cells (Tregs) and amplification of anti‐tumor immune responses. After intravenous injection, DMC@P‐Cs can efficiently accumulate at the tumor site, and local US treatment resulted in 94.73% tumor inhibition rate. In addition, there is no detectable systemic toxicity. Therefore, this study provides a highly stable and US‐controllable smart delivery system to achieve synergistical sonodynamic‐immunotherapy for enhanced colorectal cancer therapy.

## Graphical abstract

Highly stable cerasomal nano‐modulator (DMC@P‐Cs) with silicate surface and ultrasound‐controlled delivery capability is fabricated for modulating the balance of cytotoxic T lymphocytes (CTLs) and regulatory T cells (Tregs) within tumors. By locally delivering ROS and immunotherapy adjuvant to the tumors, the tumor cell‐killing function of invasive CTLs can be greatly strengthened by down‐regulating Tregs, leading to effective tumor inhibition and immune activation.

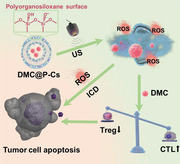

## 1 Introduction

Immunotherapy utilizing the body's immune system to battle different tumors has achieved great success in recent years.^[^ ^1^ ^]^ However, low response rates and immune‐related drug side effects hamper the clinical application of this promising therapy.^[^ ^2^ ^]^ In addition to the well‐known immune checkpoint blockade (ICB) therapies, such as PD1/PD‐L1 axis block mediated by monoclonal programmed cell death protein 1 (PD1) or programmed death ligand 1 (PD‐L1) antibodies to activate exhausted T cells,^[^ ^3^ ^]^ modulating the intratumoral balance of cytotoxic T lymphocytes (CTLs) and regulatory T cells (Tregs) is another effective strategy to enhance cancer immunotherapy, since the tumor cell killing function of infiltrating CTLs is usually inhibited by upregulated Tregs, leading to immune homeostasis and tumor progression.^[^ ^4^ ^]^ Thus, efficient cancer immunotherapy can be realized by regulating these T cell receptors mediated positive or negative signals via delivering agonistic or blocking immunotherapeutic agents to the tumor.^[^ ^5^ ^]^ However, nonspecific biodistribution in normal tissues and limited accumulation at the tumor site are often inevitable when systematically administrating the immunotherapeutic agents such as different monoclonal antibodies, cytokines, or adjuvants, thus resulting in potential immune‐related side effects and poor therapeutic efficiency.^[^ ^2^, ^6^ ^]^ To avoid these issues, hydrogels or tumor microenvironment (TME)‐responsive immune nano‐modulators have been developed for local activation of antitumor immunity.^[^ ^7^, ^8^ ^]^ However, such strategies were not suitable for invisible or inaccessible tumors or suffered from complex fabrication processes. More importantly, the heterogeneity of tumors can lead to uneven and uncontrollable drug release, which are difficult to adjust in time and in need. The controllable delivery of immunotherapeutic agents through external stimulus appears to be more advantageous, which can achieve real‐time modulation of drug release in the complicated TME.^[^ ^9^ ^]^ Therefore, alternative approaches using external stimulus to remotely and specifically activate tumor immune response and reduce immune‐related side effects are highly desired.

Among all external stimuli, ultrasound (US) is a suitable trigger for such requirements, as it is non‐invasive while with advantages of deep penetration and focusability on a certain area, facilitating accurate spatiotemporal positioning to the diseased and deep tissues and minimizing damage to the surrounding non‐targeted tissues.^[^ ^10^ ^]^ can utilize thermal, mechanical effects and sonodynamic therapy (SDT) generated reactive oxygen species (ROS) to trigger drug release from the delivery systems, such as liposomes and hybrid materials.^[^ ^10^, ^11^, ^12^ ^]^ Especially, ROS can also kill tumor cell and induce immunogenic cell death (ICD), subsequently promoting maturation of dendritic cells (DC), and finally stimulating immune responses to enhance intratumoral infiltration of cytotoxic T lymphocytes (CTLs). However, a prerequisite is the stable delivery of sonosensitiers which are usually hydrophobic porphyrin derivatives. On the other hand, for regulation of Tregs, targeting silence of transforming growth factor‐β (TGF‐β) via related siRNA or small molecular inhibitors delivering to tumor can effectively decrease TME‐resident Tregs.^[^ ^13^ ^]^ Demethylcantharidin (DMC) is a hydrophilic Tregs inhibitor that overactivates mammalian target protein complex 1 (mTORC1) signaling by specifically inhibiting PP2A activity, thereby down‐regulating the expression of forkhead box P3 (FOXP3) and reducing Tregs formation in differentiated four (CD4) T lymphocyte clusters.^[^ ^14^ ^]^ However, DMC suffers from fast metabolic rate and many side effects, including irritation of urinary system, gastrointestinal reaction, destruction of liver and kidney function, etc, which are unfavorable for treatment.^[^ ^15^ ^]^ Therefore, designing nanosystems that can load both hydrophobic sonosensitizers and hydrophilic Treg inhibitors, while controlling their release through SDT induced ROS, is highly suitable for regulating the CTLs/Tregs ratio to enhance immunotherapy.

Liposome is undoubtedly the best choice, since it has both a hydrophobic lipid bilayer and a hydrophilic core, resulting in both hydrophobic and hydrophilic drugs being efficiently loaded into the lipid bilayer and hydrophilic core, respectively.^[^ ^16^ ^]^ Drugs delivered by liposomes have been demonstrated to own better pharmacokinetic and biological distribution, and better accumulation to the target tissues.^[^ ^10^, ^17^, ^18^ ^]^ However, these liposomal systems may leak their encapsulated drug during storage or blood circulation due to their instability, resulting in impaired efficacy and an inevitable increase in adverse reactions. To address this issue, we have developed a formulation of organic–inorganic hybrid system called cerasomes,^[^ ^19^ ^]^ which not only has the double‐layer nanostructure similar to traditional liposomes, but also has a polysiloxane surface atomic layer similar to silicon dioxide, showing good biocompatibility similar to traditional liposomes but much higher morphological stability than liposomes. When the free drug is encapsulated into cerasomes, it can greatly avoid the premature release of the drug before arriving at the target sites.^[^ ^19^, ^20^ ^]^ Therefore, endowing highly stable cerasomes with US‐controlled release function may well address the afore‐mentioned issues for efficient co‐delivery of sonosensitizer and Treg inhibitor.

**Figure 1:**
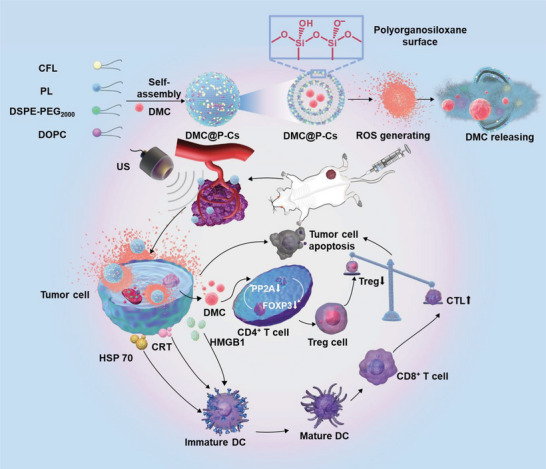
Fabrication illustration of DMC@P‐Cs and the delivery of sonosensitizers and immunosuppressants to the tumor to achieve the sonodynamic therapy of CT26 tumor combined with Immunotherapy.

Herein, a highly stable and US‐responsive cerasomal modulator DMC@P‐Cs consisting of cerasome‐forming lipid (CFL), PL conjugate, unsaturated DOPC lipid, and DMC was fabricated for US‐triggered cancer sonodynamic‐immunotherapy. The resulting DMC@P‐Cs combined with US can specifically generate ROS while trigger DMC to release from DMC@P‐Cs, which can specifically enhance the infiltration of CTLs and down‐regulate Tregs at the tumor site to counteract the tumor immune tolerance (**Figure** **1**). DMC@P‐Cs with silicate surface keep stable during the blood circulation, thus reducing DMC release and contact with the normal tissues. DMC@P‐Cs accumulates passively at the tumor site through enhanced permeability and retention (EPR) effects and can be activated to produce ROS and release DMC when US is locally applied to the tumor, in which process the unsaturated lipids DOPC in the cerasomal bilayer could be peroxidized by the produced ROS, resulting in local release of DMC. In combination with SDT, DMC@P‐Cs can trigger tumor antigen release to produce ICD through the production of ROS, promote the infiltration of CTLs in tumor, while decrease Tregs by the released DMC, thus significantly increasing the CTLs/Tregs ratio. In addition, systemic adverse reactions of this system can be ignored. Taken together, the constructed cerasomes provide a robust nanoplatform to locally deliver immunomodulator to enhance cancer immunotherapy.

## 2 Results and Discussion

### 2.1 Fabrication of DMC@P‐Cs

**Figure 2:**
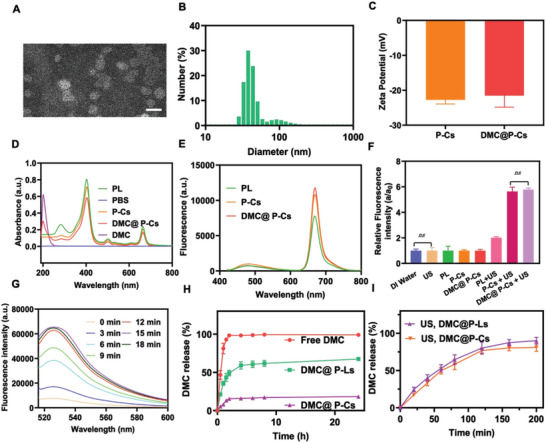
Characterization of the DMC@P‐Cs. A) TEM image of DMC@P‐Cs. Scale bar: 50 nm. B) Dynamic light scattering (DLS) measurement of DMC@P‐Cs. C) Zeta potential of P‐Cs and DMC@P‐Cs. D) UV−vis absorption spectra of PL, P‐Cs, DMC, and DMC@P‐Cs in PBS solutions. E) Fluorescence spectra of P‐Cs and DMC@P‐Cs in aqueous solutions, while PL dissolved in organic solvents were used as a control (Ex = 405 nm). F) Comparison of ROS generation ability among DI water group, US group, PL group, P‐Cs group, DMC@P‐Cs group, P‐Cs + US group, and DMC@ P‐Cs + US group (equivalent concentration of PL: 20 µg/mL). G) Fluorescence emission spectra of the DMC@P‐Cs incubated with SOSG under US irradiation for different periods (equivalent concentration of PL: 20 µg/mL, Ex: 480 nm). H) DMC release behaviors of various samples in physiological status. I) DMC release behaviors of various samples with US irradiation.

To achieve US‐controlled drug release and obtain stable cerasomes for simultaneous delivery of hydrophilic immune effector drug and hydrophobic sonosensitizer, the composition of cerasomes had to be carefully considered. A lipid mixture of CFL, DSPE‐PEG_2000_, unsaturated lipid of 1,2‐dioleoyl‐sn‐glycero‐3‐phosphocholine (DOPC), and amphiphilic pyropheophorbide‐conjugated lipid (PL) at a molar ratio of 30:5:15:50 w finally used for preparing DMC@P‐Cs using ethanol injection method in an ultrasonic water bath, through a process of self‐assembly, resulting in cerasomal nanoparticles with diameter of ≈40 nm and a high loading content for pyropheophorbide, which was chemically conjugated in PL molecules because there was not requirement for drug release to play SDT function unlike chemotherapy drugs, thus exhibiting advantage of no premature release of sonosensitizers in circulation. The chemical structures of various lipids were shown in Figure S1 (Supporting Information). DMC was encapsulated according to different ratios of drug to lipid, followed by sol−gel reaction process to form inorganosilicate network on the DMC@P‐Cs surface (Si‐OCH_2_CH_3_ + H_2_O → Si−OH + CH_3_CH_2_OH followed by 2Si−OH → Si−O−Si). To obtain optimized ratio of DMC to P‐Cs, a range of weight ratios (w/w) of DMC/P‐Cs from 1/15 to 1/2 were designed to prepare DMC@P‐Cs. The encapsulation efficiency (EE) and loading content (LC) of DMC were listed in Figure S2A,B (Supporting Information). As the drug/lipid ratio increased, EE decreased from 95.22% to 35.65% and LC increased from 6.39% to 17.95%, both satisfactory EE and LC were obtained when the drug/lipid ratio reached 1/5, which was subsequently selected to encapsulate the drug DMC to P‐Cs considering the balance between EE and LC. In addition, the morphologies and size of DMC@P‐Cs were also studied. We used transmission electron microscopy (TEM) to observe the morphologies of the prepared DMC@P‐Cs with a regular spherical structure with a size of ≈40 nm (**Figure** **2**A). The particle size of DMC@P‐Cs measured by dynamic light scattering (DLS) was 49.53 ± 7.04 nm (Figure 2B), which should be due to the hydration layer on the particle surface. In addition, we also characterized P‐Cs and DMC@P‐Ls by TEM and DLS. P‐Cs had TEM and DLS results similar to DMC@P‐Cs. However, DMC@P‐Ls exhibited a larger particle size than cerasomes, which may be due to the absence of a dense silicate network coating on its surface (Figures S3A,B and S4A,B, Supporting Information). In order to confirm the distribution of silicon in CFL, the prepared DMC@P‐Cs was characterized by scanning electron microscopy‐energy dispersive X‐ray spectrometer (SEM‐EDS). As shown in the Figure S5 (Supporting Information), C, N, O, and Si elements were uniformly distributed on DMC@P‐Cs. Since PL, DOPC, DSPE‐PEG2000, and DMC were all commercial raw materials, it can be seen from the chemical structures given in Figure S1 (Supporting Information) that these raw materials did not contain Si element, so it was proved that Si element came from CFL, and CFL and other lipids self‐assembled into DMC@P‐Cs very uniformly.

To confirm that US can promote the release of DMC from cerasomes, the morphologies of DMC@P‐Cs after US treatment were observed by TEM (US parameters:1 MHz, 1 W cm^−2^, 20% duty, 5 min) (Figure S6A, Supporting Information). After US irradiation, the cerasomes’ structure disintegrated and showed a fine sheet structure under TEM, which should be due to the oxidation of DOPC in the cerasomes’ shell. The particle size of the cerasomes with US irradiation measurement by DLS was found to decrease rapidly to only 5–6 nm (Figure S6B, Supporting Information). Different media such as saline, PBS, fetal bovine serum (FBS), Roswell park memorial institute (RPMI)−1640 medium with 10% FBS, and Dulbecco's modified Eagle's medium (DMEM) with 10% FBS, were then applied to simulate the physiological conditions, DMC@P‐Cs in these solutions all showed good dispersibility (Figure S7, Supporting Information). In addition, the particle size of DMC@P‐Cs and DMC@P‐Ls in the above‐mentioned different media were further monitored by DLS. Compared with DMC@P‐Ls, DMC@P‐Cs showed no significant changes in all media within 7 days, (Figure S8A–D, Supporting Information), exhibiting excellent colloid stability, which should be attributed to its silicate surface with strong negative *ζ* potential of −22±3.28 mV (Figure 2C). The morphological stability of DMC@P‐Cs were then evaluated via a surfactant solubilization method, DMC@P‐Ls were used as a control. After mixing with a nonionic detergent of Triton‐X 100, the particle size of DMC@P‐Cs changed only a little even when the concentration of Triton‐X 100 was as high as 45 µM, but that of DMC@P‐Ls decreased rapidly only when the concentration of Triton‐X 100 reached 5 µMm (Figure S9, Supporting Information). The absorption spectrum of DMC@P‐Cs had peaks at 405 and 202 nm, well matched to the characteristic absorption peaks of PL and DMC, respectively (Figure 2D), indicating the successful encapsulation of these two drugs in the nanoparticles. In addition, the fluorescence spectra indicated that aqueous P‐Cs were strongly fluorescent upon 405 nm excitation similar to the PL in chloroform. Furthermore, there were nearly equal fluorescence between DMC@P‐Cs and P‐Cs, suggesting that the loading of DMC into P‐Cs had no influence on the PL fluorescence (Figure 2E). In addition, we also verified the formation of silicate network structures by FTIR (Figure S10, Supporting Information). The obvious characteristic peaks at 950 and 1100 cm^−1^ corresponded to the stretching vibration absorption peaks of Si‐OH and Si‐O‐Si groups, respectively. It was worth noting that the Si‐O‐Si peak intensity was significantly greater than that of Si‐OH peak, which not only proved that silicate network structure was formed on the surface of DMC@P‐Cs, but also indicated that the structure of silicate network was relatively dense.

### 2.2 US‐Triggered ROS Generation and US‐Mediated Release of DMC

Sonosensitizer was conjugated in DMC@P‐Cs bilayer, to assess whether DMC@P‐Cs can produce ROS under US irradiation, a probe of singlet oxygen sensor green (SOSG) was used because it reacted specifically with singlet oxygen, resulting in enhancement in its green fluorescence intensity. Expectedly, there were no fluorescence change for the water, PL, P‐Cs, DMC@P‐Cs groups without US irradiation. In contrast, upon US treatment, there was two times of fluorescence enhancement for the PL+US groups as compared to the water control, indicating PL can operate as an effective sonosensitizer. Nevertheless, after self‐assembly into the P‐Cs, six times fluorescence enhancement was obtained, which should be due to the aggregation reduction of PL with the assistance of other lipids in the P‐Cs bilayer. Similar result was achieved for the DMC@P‐Cs + US group, suggesting the loading of DMC in the DMC@P‐Cs core had no effect on its ROS generation (Figure 2F). In addition, the characteristic fluorescence emission peak of the SOSG at ≈525 nm gradually increased with the extension of ultrasound time (Figure 2G). Furthermore, this effect can be further enhanced by increasing the concentration of DMC@P‐Cs (equivalent concentration of PL: 20, 50, 80,150 µg mL^−1^) (Figure S11A, Supporting Information). With the increase of ultrasonic power and duty cycle, the fluorescence of SOSG became stronger and stronger (Figure S11B,C, Supporting Information). Therefore, DMC@P‐Cs showed well ultrasound responsibility, which were well suitable for controlled local drug release.

Good stability of cersasomal DMC@P‐Cs enabled its capability to reduce premature drug leakage, which was beneficial for the drug delivery during blood circulation. However, after reaching the tumor, it was expected that the drug can be controlled release as needed. Therefore, we further investigated whether the SDT‐mediated ROS can control the release of drugs from DMC@P‐Cs. Expectedly, DMC@P‐Cs at physiological temperature (37 °C, PBS, pH 7.4) exhibited very slow release rate with only 18.2±1.4% within 24 h, whereas the drug‐loaded liposomes (DMC@P‐Ls) and free DMC groups released 67.4±2.1% and almost 100% of the drugs within 24 h, respectively (Figure 2H). The release difference between the DMC@P‐Cs and DMC@P‐Ls should be due to the silicate network structure of DMC@P‐Cs, which restricted the movement of the bilayer membrane carbon chains, resulting in decreased fluidity of the membrane and the reduced drug release rate.^[^ ^21^ ^]^ The US‐triggered DMC release from DMC@P‐Cs was then further investigated. It was found that the DMC release rate was positively correlated to the duration and intensity of ultrasound. The higher of these parameters, the quicker release of the encapsulated drugs (Figure S12, Supporting Information). The release rate of DMC from DMC@P‐Cs was significantly accelerated after US irradiation (1.0 MHz, 1 W cm^−2^, 20% duty, 5 min), reaching ≈(76.8±3.5)% within 120 min, which was about five times higher than that of DMC@P‐Cs without US treatment (≈(15.5±1.56)%). It was worth mentioning that cerasomal DMC@P‐Cs had better stability than liposomal DMC@P‐Ls, but it exhibited almost the same drug release rate as the liposome when promoting by ROS (Figure 2I), demonstrating its significant advantages as ultrasound responsive drug carriers. In this process, US acted as a switch that triggered the release of the drug. To demonstrate that ROS production was the mechanism that triggered drug release, we irradiated DMC@Cs without PL with US (1.0 MHz, 1 W cm^−2^, 20% duty, 5 min, sonicated at 0, 1, 2 h) and compared with DMC@P‐Cs. The results demonstrated that there was no significant change in the drug release from DMC@Cs with or without US (Figure S13, Supporting Information). This further confirmed the mechanism of the increased drug release should be that DOPC in the DMC@P‐Cs bilayer was peroxidized by ROS, leading to generation of drug releasing channels in the bilayer and thus causing rapid release of drug from the carrier cores.

### 2.3 Cellular Uptake, US‐Induced Intracellular ROS Generation, and DMC@P‐Cs Mediated Cytotoxicity In Vitro

**Figure 3:**
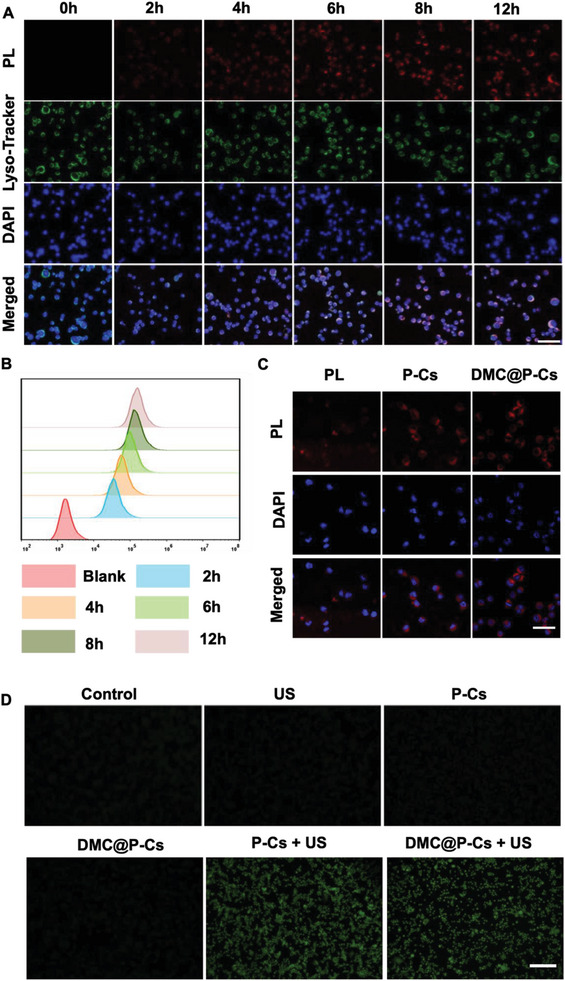
In vitro cellular uptake and ROS production capacity of DMC@P‐Cs. A) The time‐dependence of CLSM images of CT26 cells after incubation with DMC@P‐Cs (equivalent concentration of PL: 20 µg mL^−1^). Scale bar: 40 µm. B) Flow cytometry analysis for cellular uptake of CT26 cells incubated with DMC@P‐Cs at the PL concentration of 20 µg mL^−1^ for different time. C) CLSM images of CT26 cells after incubation with various materials: PL, P‐Cs, and DMC@P‐Cs (equivalent concentration of PL: 20 µg mL^−1^). Blue signals indicated the location of cell nucleus, red signals indicated the internalization of DMC@P‐Cs, and green signals indicated the lysosomes stained with lysotracker green. Scale bar: 40 µm. D) Fluorescence images of CT26 cells treated in various ways: Control (without any treatment), US, P‐Cs, DMC@P‐Cs, P‐Cs + US, and DMC@P‐Cs + US (equivalent concentration of PL: 20 µg mL^−1^). Scale bar: 200 µm.

To investigate the cellular uptake of DMC@P‐Cs, CT26 cells were incubated with DMC@P‐Cs for various periods and observed by confocal laser scanning microscope (CLSM). Red fluorescence signals of DMC@P‐Cs derived from PL in the nanoparticle bilayer were gradually enhanced with the increase of incubation time, showing maximum uptake at 8 h, after the cell lysosomes were stained with lysotracker green, the pink fluorescence obtained by the overlapping of PL and lysosomal green clearly showed that DMC@P‐Cs was mainly located in the cytoplasm (**Figure** **3**A), suggesting a time‐dependent endocytosis behavior of DMC@P‐Cs.^[^ ^22^ ^]^ The results of flow cytometry analysis were consistent with the CLSM results (Figure 3B), showing quick uptake in the first 2 h, followed by gradual increase in the next 6 h. We conducted a semi‐quantitative analysis on the data in Figure 3A,B, and the experimental results were shown in the Figure S14 (Supporting Information). Semi‐quantitative analysis of the two experiments both showed no significant difference (*ns*) between 8 and 12 h. In addition, compared with P‐Cs, the cellular uptake of DMC@P‐Cs was not significantly altered, indicating that the drug loading did not affect its uptake (Figure 3C). Non‐fluorescent DCFH‐DA probe was used to directly monitor the ROS production of DMC@P‐Cs in CT26 cells under US irradiation, which can be converted into green fluorescent 2, 7‐dichlorofluorescein (DCF) after oxidation. As shown in Figure 3D, there was no fluorescence for the US, P‐Cs, or DMC@P‐Cs only groups, suggesting no intracellular ROS generation similar to the control group. In contrast, ROS levels in the “P‐Cs + US” group and the “DMC@P‐Cs + US” group were significantly increased, and strong green fluorescence could be seen in DCF channel, which was in good agreement with the ROS detection results in solution (Figure 2F). These findings demonstrated the efficacy of DMC@P‐Cs to be a high‐performance nanoacoustic sensitizer. In addition, we conducted a semi‐quantitative analysis of the green fluorescence signal in Figure 3D, and the results further proved that PL in DMC@P‐Cs could efficiently produced ROS under the irradiation of US (Figure S15, Supporting Information).

**Figure 4:**
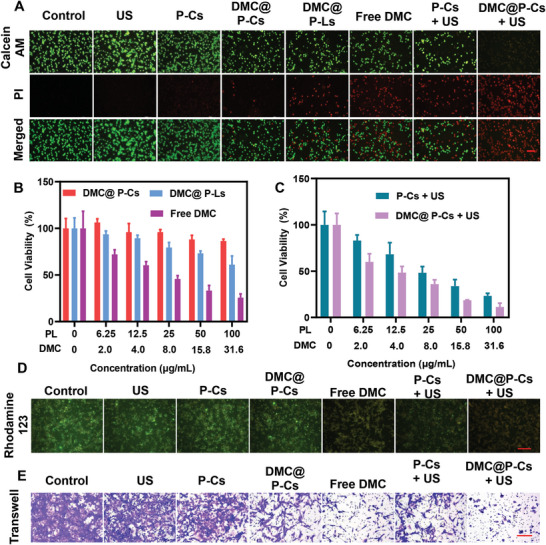
Experiments on the killing ability and mechanism of DMC@P‐Cs on CT26 cells. A) Fluorescence images of CT26 cells stained with the LIVE/DEAD activity/cytotoxicity kit. Scale Bar: 100 µm. B) Viability of CT26 cells incubated with different concentrations of DMC@P‐Cs, DMC@P‐Ls and Free DMC. C) Viability of CT26 cells treated in different ways: P‐Cs + US, DMC@P‐Cs + US. D) Fluorescence images of Rodamine 123‐labeled CT26 cells treated in the same way as the above‐mentioned evaluation of intracellular ROS production. Scale bar: 200 µm. E) The invasion ability of CT26 cells was analyzed by Transwell. Scale bar: 200 µm.

Inspired by the DMC@P‐Cs's superior properties in cellular uptake and ROS generation, its SDT‐mediated killing effect was assessed on CT26 cancer cells. To ensure the biosafety of carrier P‐Cs, Human Umbilical Vein Endothelial Cells (HUVECs) were first selected for biocompatibility analysis. The viability of HUVECs was determined by MTT and LIVE/DEAD activity/cytotoxicity methods. After 24 h treatment with P‐Cs (0‐200 µg mL^−1^), HUVECs showed no obvious cytotoxicity (Figures S16 and S17, Supporting Information). In particularly, there was still over 90% cells alive at the high concentration of 200 µg mL^−1^, confirming an acceptable biocompatibility for potential clinical application. Biosafety of external stimulus of US was also investigated, exhibiting no significant adverse effects on HUVEC's cell viability after irradiated by US for 1–5 min at both 20% and 50% duty cycles (1 W cm^−2^), suggesting a satisfactory biosafety profile for irradiation with these exogenous energies during therapy (Figure S18, Supporting Information). Then Live/Dead cell viability/cytotoxicity kit was applied to evaluate the US‐triggered SDT cytotoxicity on CT26 cells mediated by DMC@P‐Cs, showing green (Calcein AM)/red (PI) fluorescence, respectively (**Figure** **4**A). No obvious cytotoxicity existed for US and P‐Cs groups with only green fluorescence, nonetheless, slightly red fluorescence spots were appeared in the DMC@P‐Ls group, which should be due to the encapsulated DMC drug with high cytotoxicity as exhibited in the free DMC group. Therefore, DMC loaded in highly stable cerasomes was necessary to reduce unwanted side effects. When irradiated by US, both P‐Cs and DMC@P‐Cs groups showed obvious cytotoxicity to the CT26 cells, and the red fluorescence in the P‐Cs was obviously weaker than DMC@P‐Cs group, which revealed not any green fluorescence. Such high cell killing effect should be attributed to both the SDT effect of PL and the released DMC in DMC@P‐Cs when applied US irradiation.

The cytotoxicity of DMC@P‐Cs to CT26 tumor cells with or without US irradiation was further quantified by the MTT assay. Likewise, DMC@P‐Cs had the least cytotoxicity as compared to DMC@P‐Ls and free DMC at all tested concentrations (Figure 4B). The encapsulation of DMC in DMC@P‐Ls and DMC@P‐Cs can significantly reduce its toxicity, where the latter carrier was obviously more advantageous than the former one, which was undoubtedly owing to the higher stability of cerasomes with reduced drug leakage. By contrast, obvious cell viability decrease was observed when treated with both P‐Cs + US and DMC@P‐Cs + US (Figure 4C), the higher cell killing effect of DMC@P‐Cs + US than P‐Cs + US should be due to the SDT‐triggered released DMC, showing concentration‐dependence. There was nearly 87.8% cell viability reduce at the concertation of 100 ug mL^−1^ PL for DMC@P‐Cs treated cells after US irradiation, again demonstrating both the high stability and well US responsiveness of DMC@P‐Cs, which was well suited for site‐specific drug delivery.

### 2.4 Assessment of Mitochondrial Membrane Potential and Tumor Cell Invasiveness

When mitochondrial membrane potential decreased, mitochondrial membrane permeability increased, and mitochondrial pro‐apoptotic factors (e.g., Cyt C, AIF, SMAC/DIABLO, HTRA2/OMI, ENDOG) were released into the cytoplasm. After Cyt C was released into the cell, it interacted with Apaf‐1 and formed an apoptotic complex with the help of ATP and dATP, which recruited and activated Pro‐Caspase 9 to form holoenzyme Caspase 9. Holoenzyme Caspase 9 further activated Caspase 3 and Caspase 7, initiated the Caspase cascade, cleaved over 100 substrates such as α‐tubulin, Actin, PARPA, and Lamin in cells, and ultimately led to apoptosis.^[^ ^23^ ^]^ To further reveal the mechanism of DMC@P‐Cs mediated cell killing effect, mitochondrial membrane potential of CT26 cells with different treatments was evaluated by Rhodamine 123 (Rh 123) kit, which can enter into or leach out from cell mitochondria with intact or abnormal membrane potential, thus showing strong or weak yellow‐green fluorescence, respectively. After different treatment for 6 h, US, P‐Cs, and DMC@P‐Cs groups all showed strong yellow‐green fluorescence as the control group (Figure 4D). In contrast, obviously weaker fluorescence was observed for both free DMC and P‐Cs + US groups, suggesting cell damages by cytotoxic DMC or SDT induced ROS in these groups. Unsurprisingly, the most pronounced decrease in green fluorescence was appeared in the DMC@P‐Cs + US group, which combined both killing effects of free DMC and P‐Cs + US, thus resulting in the maximum decrease in intracellular mitochondrial membrane potential.

A Transwell assay was further applied to examine the CT26 cells invasion after different treatments (Figure 4E), where US and P‐Cs groups showed similar cell invasion to the control group, while DMC@P‐Cs and free DMC suppressed the cell invasion by 36.8% and 53.2%, respectively, indicating that the cytotoxicity of DMC can be greatly reduced when loaded in the P‐Cs. When combination with US, P‐Cs + US exhibited a stronger inhibited effect (37.4%) than both P‐Cs only and US only groups. Similarly, the strongest inhibited effect on tumor cell invasion was achieved in DMC@P‐Cs + US group with 76.9% inhibition rate, nearly equivalent to a 4.35‐fold reduction in invasion compared to the control (Figure S19, Supporting Information).

### 2.5 SDT‐Triggered Immunogenic Cell Death Mediated by DMC@P‐Cs

**Figure 5:**
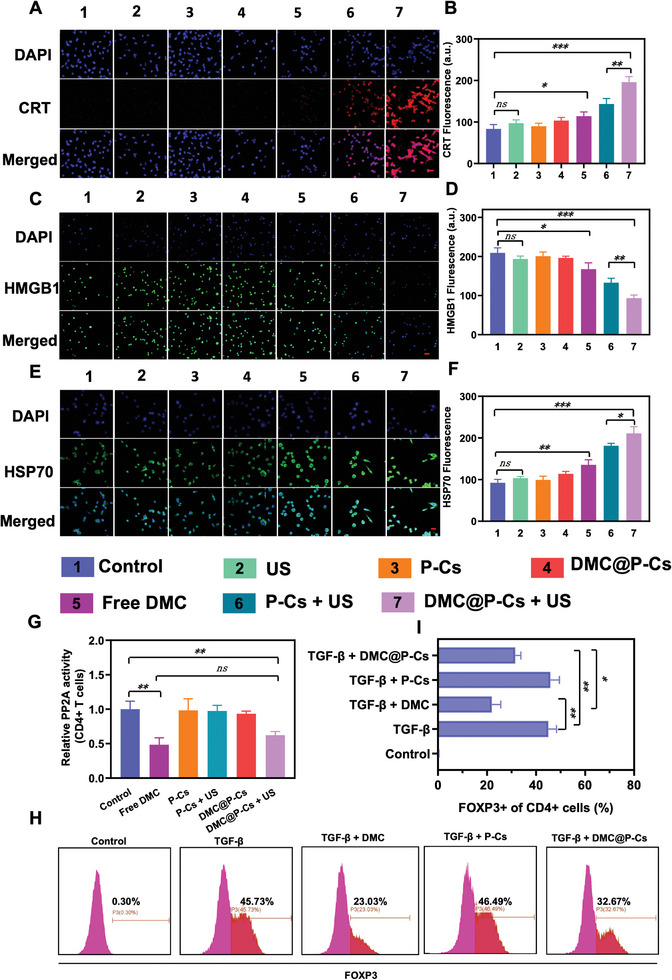
SDT‐Triggered ICD mediated by DMC@P‐Cs and inhibition of Tregs formation by DMC@P‐Cs combined with US. A) CRT exposed on CT26 cell surface after different treatments as observed by CLSM. Scale bar: 20 µm. B) Quantification of CRT signal intensity for different groups in (A). Data were presented as mean ± SD (*n* = 3). C) HMGB1 released from CT26 cells after different treatments as observed by CLSM. Scale bar: 20 µm. D) Quantification of HMGB1 signal intensity for different groups in (C). Data were presented as mean ± SD (*n* = 3). E) HSP70 exposed on CT26 cell surface after different treatments as observed by CLSM. Scale bar: 20 µm. F) Quantification of HSP70 signal intensity for different groups in (E). Data were presented as mean ± SD (*n* = 3). G) PP2A activity of CD4^+^ T cells after various treatments with control, free DMC, P‐Cs, P‐Cs + US, DMC@P‐Cs, and DMC@P‐Cs + US for 8 h. H) Flow cytometry analysis of intracellular FOXP3 expression of CD4^+^ T cells after incubation with different treatments for 3 days in the presence of anti‐CD3 and CD28. I) Quantification of CD4^+^FOXP3^+^ T cells after incubation of naive CD4^+^ T cells with different treatments for 3 days in the presence of anti‐CD3 and CD28.

In addition to cell viability and cell invasion detection for DMC@P‐Cs, immunogenic cell death (ICD) was another important issue associated with tumor therapy, which can trigger the maturation of dendritic cells (DC) by dying tumor cells released damage associated molecular patterns (DAMPs), resulting in enhanced immune response.^[^ ^24^ ^]^ The common biomarkers of calreticulin (CRT), intracellular high‐mobility group protein B1 (HMGB1), and cell‐surface heat‐shock proteins 70 kDa (HSP70) were then detected. For CRT which transferred from endoplasmic reticulum to cell surface when tumor cells experienced ICD, it was found that remarkably stronger red fluorescence was only observed in both “P‐Cs + US” and “DMC@P‐Cs + US” groups, with mean fluorescence intensity (MFI) of CRT expression increase on tumor cells by respective 1.72 and 2.35 folds as compared with the blank control (**Figure** **5**A,B), and the higher expression of CRT in the “DMC@P‐Cs + US” than in “P‐Cs + US” groups may benefit from DMC‐mediated cell‐killing effect, as evidenced by the slight increase of MFI in the “Free DMC” group, whereas US only, P‐Cs or DMC@P‐Cs exhibited no CRT expressions, again demonstrating the safety of these groups. For HMGB1 which acted in a cytokine‐like manner to bind antigen presenting cells (APCs) released to the extracellular space and induced protective immunity, similar results were also observed (Figure 5C,D). Only US, P‐Cs or DMC@P‐Cs treated tumor cells showed no significant release of HMGB1 as the control group, and the HMGB1 levels in “P‐Cs + US” and “DMC@P‐Cs + US” groups decreased by 36% and 55%, respectively, suggesting the loss of both nuclear and plasma membrane integrity in these groups. For HSP70 which would be exposed on the membrane of cells undergone ICD was also detected to be highly upregulated in both “P‐Cs + US” and “DMC@P‐Cs + US” groups, with respectively 1.96 and 2.28 folds increase compared to the control group. The difference of these two groups was ascribed to DMC, which can also induce a moderate HSP70 over‐expression (Figure 5E,F). We also explored the ICD effect caused by PL group and PL + US group according to the previous process, and all the experimental conditions and confocal shooting parameters were the same as before. The relevant experimental results were shown in the Figure S20 (Supporting Information). PL could not cause ICD effect after only co‐incubation with CT26, but PL could cause slight ICD effect after US irradiation, which was consistent with the results in Figure 2F. The experimental results further proved that PL can produce ROS and induce ICD in CT26 cells after US irradiation.

Collectively, CRT translocation, HMGB1 release and HSP70 expression can be effectively triggered by DMC@P‐Cs combined with US. During this process, there were not only ROS generation, but also ROS controlled released DMC, both of which performed synergistic effect, thus causing the highest ICD, which showed great potential in enhancing immune stimulatory effects for tumor elimination.

### 2.6 Inhibition of Tregs Formation by DMC@P‐Cs Combined with US

There were a variety of immunosuppressive cells in tumors, which will attenuate the tumor‐killing function of infiltrating lymphocytes and affect the effect of immunotherapy.^[^ ^25^ ^]^ Tregs was one of the most abundant immunosuppressive cells in tumors, its down‐regulation will greatly augment the immunotherapy. DMC had been reported to be an effective immunomodulator to decrease Tregs in TME via a sequential process of inhibiting activity of PP2A, downregulating expression of forkhead box P3 (FOXP3) and decreasing formation of Tregs from CD4^+^ T lymphocytes. To evaluate the function of DMC@P‐Cs on reprogramming the Tregs in vitro, CD4^+^ T cells were first collected from spleens of the healthy mice and then treated with DMC@P‐Cs in the presence or absence of US, followed by PP2A activity detection by the Ser/Thr phosphatase assay (Figure 5G). P‐Cs without DMC and free DMC were used as the controls. Similar to the free DMC with inhibition rate of ≈49.00%, it was clearly observed that DMC@P‐Cs + US obviously reduced the PP2A activity by 37.38%. While DMC@P‐Cs without US irradiation remained unchanged in PP2A activity as the P‐Cs, P‐Cs + US, and control groups. It was apparent that DMC@P‐Cs can stably retain DMC and effectively generate ROS upon US treatment to control release of the encapsulated DMC, thus causing effective PP2A inhibition. Next, in the presence of anti‐CD3, anti‐CD28, naive CD4^+^ T cells were treated with DMC@P‐Cs and TGF‐β, which was an immunosuppression mediator to induce FOXP3 expression (Figure 5H,I). About 45.73% enhancement of the intracellular FOXP3 expression was observed for the TGF‐β treated group, which was decreased to 23.03% when combined with free DMC, indicating inhibited effect of DMC on FOXP3 expression. In addition, TGF‐β + P‐Cs had similar FOXP3 expression to the TGF‐β group, indicating the carrier had not effects on FOXP3 expression. Then DMC@P‐Cs were pre‐irradiated by US and used for the treatment. It was found that FOXP3 expression decreased to 32.67% when treated with TGF‐β + DMC@P‐Cs + US, which should be attributed to the ultrasound controlled released DMC from DMC@P‐Cs. Therefore, DMC@P‐Cs held the potential to specifically decrease Tregs differentiation from CD4^+^ T cells when combination with US.

### 2.7 Biocompatibility and Biodistribution of DMC@P‐Cs In Vivo

The biocompatibility of DMC@P‐Cs in vivo was first evaluated via hemolysis assay of red blood cells (RBCs). Positive control of deionized water (DI) and negative control of PBS were used. After incubating RBCs with various concentration of DMC@P‐Cs for 8 h, it was observed that the hemolysis rate showed concentration‐dependent increase, however, it was still no below 8% even at a high DMC@P‐Cs concentration of 300 µg mL^−1^ (Figure S21, Supporting Information). DMC@P‐Cs was then intravenously injected into the healthy mice to further investigate its potential toxicity by blood serum analysis. Blood was collected at 1, 2, 7, 14 days and the blood indicators of RBC, WBC, PLT, MCV, HGB, HCT, MPV, and PDW were tested, showing no apparent change compared to the control (Figure S22, Supporting Information). These results indicated good hemocompatibility of DMC@P‐Cs for blood circulation.

**Figure 6:**
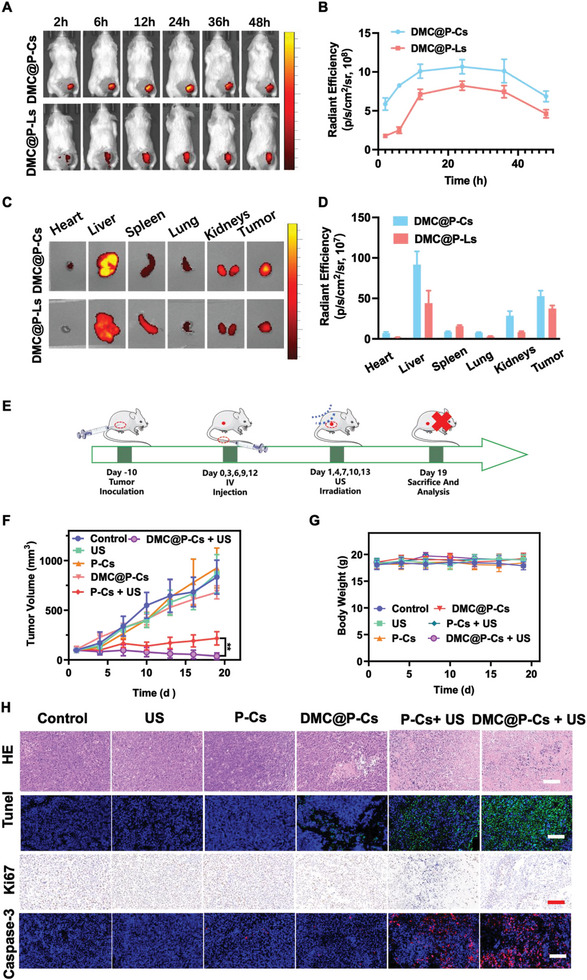
Evaluation of the anti‐tumor effect in vivo. A) In vivo NIR fluorescence images of CT26 tumor‐bearing mice after i.v. administration of DMC@P‐Cs/DMC@P‐Ls (PL: 4mg/kg) (n=3). B) Quantification of NIR signals of tumor sites of CT26 tumor‐bearing mice at different time‐points showed in (A) C) Fluorescence images of major organs and tumors from mice at 48 h post injection of DMC@P‐Cs/DMC@P‐Ls. D) Quantitative analysis of fluorescence intensity of tumors and main organs showed in (C). E) Schematic diagram of the treatment route. F) Tumor volume change after different treatments. G) Body weight change of mice in the 19 days' treatment. H) H&E staining, TUNEL staining, Ki67 immunohistochemical staining and Caspase‐3  immunofluorescence staining of the tumor tissue slices after different treatments (PBS, US, P‐Cs, DMC@P‐Cs, P‐Cs+ US, and DMC@P‐Cs + US). Scale bar: 100 µm.

Due to the silicate network structure on the surface, DMC@P‐Cs had good stability and can reduce unnecessary drug leakage during its blood circulation. Moreover, the tumor enrichment efficiency was another key issue affecting its further therapeutic effect, in which blood circulation time was an important factor. After intravenous (*I.V*.) injecting DMC@P‐Cs into healthy mice, the pharmacokinetics of DMC@P‐Cs was assessed by measuring the ultraviolet absorption of DMC at different times post‐injection (Figure S23, Supporting Information). The results showed that the half‐life t_1/2_ of DMC was 4.5 h. This duration was later conducive to the accumulation of DMC@P‐Cs at the tumor site through EPR effect. Subsequently, we used the IVIS system to trace DMC@P‐Cs and DMC@P‐Ls injected into mice through PL fluorescence signal (**Figure** **6**A). Both the cerasomes and liposomes showed the highest tumor accumulation at 24 h after injection, but the accumulation of cerasomes was significantly higher than that of liposomes, possibly due to the silicon‐containing shell of the cerasomes (Figure 6B). Furthermore, the fluorescence imaging of tumor can still be clearly observed even at 48 h, providing enough time for guiding US irradiation at the tumor site. Then, the biological distribution of DMC@P‐Cs and DMC@P‐Ls was studied by detecting the 48 h in vitro fluorescence imaging of major organs. The DMC@P‐Cs group showed stronger fluorescence in tumor tissues (Figure 6C,D), which further demonstrated that the system had significant tumor accumulation and was very suitable for efficient drug administration. Subsequently, we extracted DMC from various organs and quantified it (Figure S24, Supporting Information). The results further proved that DMC@P‐Cs was more conducive to the enrichment of DMC in tumors.

### 2.8 In Vivo Sonodynamic‐Immunotherapy Effect Mediated by DMC@P‐Cs

DMC@P‐Cs possessed high stability, US‐controlled efficient ROS generation, and drug release in vitro, together with its excellent tumor accumulation in vivo, which promoted us to further evaluate DMC@P‐Cs mediated sonodynamic‐immunotherapy effects on a CT26 tumor model. When the tumor volume grew to ≈100 mm^3^, mice were randomly allocated into six groups (n = 5 for each group): (1) PBS, (2) US, (3) P‐Cs, (4) DMC@P‐Cs, (5) P‐Cs + US, and (6) DMC@P‐Cs + US. Different agents were then administrated on day 0, 3, 6, 9, and 12 at PL dose of 4 mg kg^−1^ and DMC dose of 1.27 mg kg^−1^, US irradiations were conducted at 24 h post‐injection (1 MHz, 1 W cm^−2^, 50% duty cycle, 5 min) (Figure 6E), tumor volume and body weight were periodically measured every 3 days (Figure 6F,G). “US” and “P‐Cs” groups showed rapid tumor growth from ≈100 to ≈800 mm^3^ as the PBS control group, demonstrating the safety of US and the nanoparticle itself. It should be noted that DMC@P‐Cs exhibited negligible anticancer therapeutic effect, again demonstrating the high stability of DMC@P‐Cs with very few drug leakage. By contrast, with US irradiation, “P‐Cs + US” treated mice showed significantly slow tumor growth, with inhibition rate of 73.95% on day 19, which was attributed to the PL‐mediated ROS generation. In sharp contrast, the best therapeutic efficacy was achieved in “DMC@P‐Cs + US” treated group, tumor volume in this group displayed a unique reduction over time, showing high inhibition rate of 94.89% after 19 days of treatment (Figure 6F). This result was expected since DMC@P‐Cs + US not only generated ROS, but also promoted DMC release from the nanoparticles, both of which contributed to achieving the best therapeutic effect. At the end of therapy, mice in different groups were sacrificed and tumors were collected, similar therapeutic trends were also observed from tumor sizes and weights (Figures S25 and S26, Supporting Information). In addition, nearly no significant change in mice body weights was occurred in different groups, demonstrating the good safety and tolerance of DMC@P‐Cs for cancer therapy (Figure 6G). Considering that the drug was mainly metabolized by liver and kidney after injection into the body, blood biochemistry of mice in different groups was tested after treatment (Figure S27, Supporting Information). Alanine aminotransferase (ALT) and aspartate aminotransferase (AST) were important indicators of liver function, and creatinine (CREA) and urea nitrogen (UREA) were important biochemical indicators of renal function. Compared with the Control group, blood biochemistry of each treated group did not change significantly, indicating that liver and kidney functions were not damaged.

### 2.9 Histological Analysis

To reveal the mechanism of antitumor effect of DMC@P‐Cs, histological analysis was performed for the tumors in different treated groups (Figure 6H). Hematoxylin and eosin (H&E) staining indicated little nuclei decrease in the US, P‐Cs, or DMC@P‐Cs‐treated mice, exhibiting no significant difference to the group treated with PBS. Notably, nuclear shrinkage and nuclei karyorrhexis were observed in the tumors treated with P‐Cs + US and DMC@P‐Cs + US, suggesting serious histopathological damage of tumor tissues. Such results were further confirmed by the antigen‐Ki67 staining, also showing positive tumor nuclei decrease with the order of PBS ≈ US ≈ P‐Cs ≈ DMC@P‐Cs < P‐Cs + US < DMC@P‐Cs + US. Additionally, TUNEL assay revealed the highest level of apoptotic cells (green fluorescence) were appeared in DMC@P‐Cs + US treated group, whereas US, P‐Cs, or DMC@P‐Cs showed no or a small number of apoptotic cells, which was finally verified by caspase‐3 expression detected by caspase‐3 antibody, where DMC@P‐Cs + US again had the strongest red fluorescence from Cy3‐labeled caspase‐3 antibody. All these results suggested DMC@P‐Cs can stably delivery DMC to tumor tissue and specifically destroy tumors when activated by US. After different treatments for 19 days, histological sections and H&E staining were performed for the vital organs of heart, liver, lung, spleen, and kidney collected from the treated mice. There were little pathological abnormalities or destructions in all groups compared to the PBS control group (Figure S28, Supporting Information), further verifying the good biocompatibility of DMC@P‐Cs.

### 2.10 DMC@P‐Cs Mediated Antitumor Immune Response

**Figure 7:**
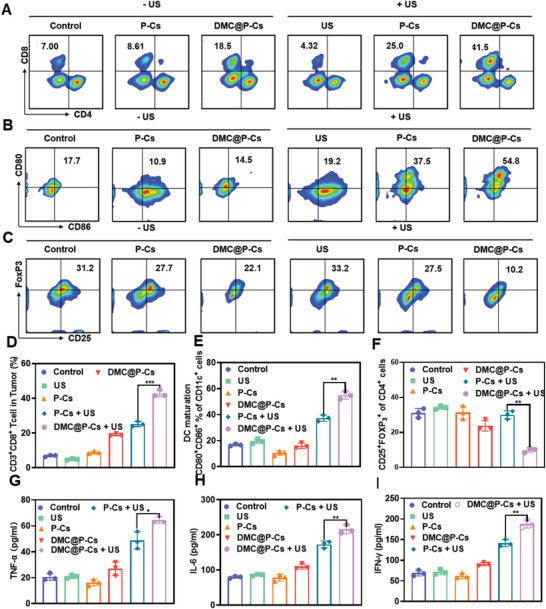
DMC@P‐Cs mediated antitumor immune response. A) Percentages of CD4^+^/CD8^+^ T cells within the tumors of mice receiving the indicated treatments. B) Representative flow cytometric plots and the percentages of mature DCs (CD11c^+^CD80^+^CD86^+^) in tumor‐draining lymph nodes. C) Percentages of FOXP3^+^ T cells within the tumors of mice receiving the indicated treatments. Data were presented as mean ± SD (*n* = 3). D) Quantification the percentage of CD8^+^ T cells shown in (A). Data were presented as mean ± SD (*n* = 3). E) Quantification the percentage CD80^+^CD86^+^cells shown in (B). Data were presented as mean ± SD (*n* = 3). F) Quantification the percentage CD25^+^FOXP3^+^cells shown in Figure 7C. Data were presented as mean ± SD (*n* = 3). Cytokine levels of G) IFN‐γ, H) IL‐6, and I) TNF‐α in the serum collected from CT26 tumor‐bearing mice after different treatments at day 13 (*n* = 3).

Antitumor immunotherapeutic mechanism of DMC@P‐Cs combined with US was finally investigated. The numbers of CD8^+^ T cells and helper T cells (CD4^+^ T cells) in tumors of different treated groups were first evaluated via flow cytometry (**Figure** **7**A,D) and the gating strategy was shown in Figure S29 (Supporting Information). There were significant increases in CD3^+^CD8^+^ T cells for both DMC@P‐Cs + US and P‐Cs + US treated groups (42.7±1.9% and 25.2±1.5%), which were respective nearly 6.29 times and 3.71 times of the control group, whereas no obvious change in CD3^+^CD8^+^ T cells occurred in the US group or P‐Cs group. Notably, DMC@P‐Cs + US mediated sonodynamic‐immunotherapy recruited 1.70 times of CD3^+^CD8^+^ T cells compared to P‐Cs + US mediated sonodynamic therapy, which should be due to the successful release of DMC encapsulated in DMC@P‐Cs after ultrasound irradiation. Thus, DMC@P‐Cs‐mediated sonodynamic‐immunotherapy caused increased infiltration of CD8^+^ T cells in TME, resulting in much higher CD8^+^/CD4^+^ ratio (2.10) as compare to the control group (0.12).

In the therapeutic process, the generated tumor‐related antigens would trigger dendritic cells (DCs) to maturation in draining lymph nodes, enhancing antigens presentation and activation of T cells.^[^ ^26^ ^]^ To access the effect of DMC@P‐Cs with or without US on the maturation of DCs, tumor‐draining lymph nodes after different treatments were collected and detected by flow cytometry (Figure 7B,E). DMC@P‐Cs group with ultrasound showed the highest mature DCs level of 54.9 ±2.95%, nearly 4 times of DMC@P‐Cs group without ultrasound. Similar results were also obtained for the P‐Cs treated groups, with 3.4 times increased number of mature DCs before and after ultrasound irradiation, demonstrating the SDT effect on triggering DCs maturity. Especially, DMC@P‐Cs + US group showed ≈ 1.5 times mature DCs number of P‐Cs + US group, confirming both ROS and DMC mediated cytotoxicity contributed to the maturation of DCs. In contrast, there was no obvious changes for other groups, showing good biocompatibility and biosafety of these groups without US irradiation.

The level of Treg cells in tumor tissues were further evaluated with flow cytometry to evaluate the function of DMC (Figure 7C,F). DMC@P‐Cs treated group without US exhibited slightly decrease in CD4^+^CD25^+^FOXP3^+^ Tregs cells numbers in CD3^+^ T cells (22.1%) compared to PBS control (31.07±2.60%) and P‐Cs groups (27.7±3.12%), which was probably due to small amount of DMC released from DMC@P‐Cs. In sharp contrast, the amount of Tregs in DMC@P‐Cs + US treated group significantly decreased to 9.9±1.35%, significantly lower than that in P‐Cs + US and DMC@P‐Cs without US. Interestingly, P‐Cs treated group showed nearly no change in Tregs amounts before and after ultrasound, suggesting the down‐regulation of Tregs was owing to the DMC, which was controlled release from the stable DMC@P‐Cs with irradiation by US. To sum up, DMC mediated PP2A inhibition and SDT induced CD8^+^ T cells infiltration in tumor tissue, both of these functions were well combined in stable and ultrasound‐responsive DMC@P‐Cs, thus resulting in significantly enhanced ratio of CD8^+^ T cells to Tregs, and ultimately augmenting tumor immunotherapy.

Moreover, immune‐relevant cytokines of tumor necrosis factor‐α (TNF‐α), interleukin‐6 (IL‐6), and interferon‐γ (IFN‐γ) after different treatments were evaluated (Figure 7G–I), all showing serum levels order of PBS control ≈ US ≈ P‐Cs < DMC@P‐Cs < P‐Cs + US < DMC@P‐Cs + US, where these cytokines were nearly 3.12, 2.70, and 2.73‐fold increase in DMC@P‐Cs + US as compared to PBS control groups, respectively. In addition, compared with P‐Cs + US, DMC@P‐Cs + US showed a better effect of promoting inflammatory factors. Taken all the above results together, related therapeutic mechanism could be deduced: ROS generated from P‐Cs + US can ablate tumor and cause ICD, while DMC@P‐Cs + US possessed additional function of triggering release of immunomodulators DMC in situ at the tumor area, further induced multiple pro‐inflammatory cytokines release, thus resulting in the most significant promotion of DC maturation and the highest activation of anti‐tumor immune response, showing great potentials in enhancing cancer immunotherapy.

## 3 Conclusion

We successfully fabricated a cerasomal nano‐modulator DMC@P‐Cs with sonosensitiers conjugated in the bilayer and immune‐modulator encapsulated in the core for achieving ultrasound controlled synergistic sonodynamic‐immunotherapy of cancer. Highly stable DMC@P‐Cs with silicate surface can make DMC retain in its core area, greatly reduced premature release during the blood circulation. More importantly, with ultrasound specific irradiation at the CT26 tumor tissue, DMC@P‐Cs effectively generated ROS to not only ablate tumors, induce ICD and promote DCs maturation, but also oxidize unsaturated phospholipids in the DMC@P‐Cs bilayer to open the drug release channel, causing ultrasound responsive in situ DMC release, which can significantly reduce Tregs, thereby promoting the infiltration of CTLs in tumor, thus achieving up‐regulation of CTLs/Tregs ratio. Moreover, the drug delivery and therapeutic functions in this system were specifically activated by ultrasound, effectively reduced the immune‐modulator related side effects, demonstrating to be a robust strategy for enhance tumor immunotherapy. In addition, P‐Cs possessing high stability and ultrasound‐responsiveness can also operate as a universal platform to deliver other immunomodulator molecules specifically to the tumor to meet different immunotherapy needs.

## 4 Experimental Section

### Chemicals and Materials

Cerasome forming lipid (CFL) was synthesized with reference to the literature.^[^ ^27^ ^]^ 1,2‐Distearoyl‐sn‐glycero‐3‐phosphocholine (DSPC), 1,2‐distearoyl‐sn‐glycero‐3‐phosphoethanolamine‐*N*‐[methoxy (poly‐ethylene glycol)−2000] (DSPE‐PEG_2000_), and 1,2‐dioleoyl‐sn‐glycero‐3‐phosphocholine (DOPC) were the products of Xi'an Ruixi Biological Technology Co., Ltd. Pyropheophorbide‐lipid (PL) was purchased from Xi'an Qiyue Biological Technology Co., Ltd. Demethylcantharidin (DMC) was purchased from Sigma‐Aldrich. Mitochondrial Membrane Potential Assay Kit with Rhodamine 123, LIVE/DEAD viability/cytotoxicity kit, and Reactive Oxygen Species Assay Kit (DCFH‐DA) were purchased from Beyotime Biotechnology. Lyso‐tracker Green and crystal violet (G1059) were purchased from Solarbio Scientific. Alexa Fluor@594 anti‐HMGB1, Alexa Fluor@647 anti‐CRT, Alexa Fluor@488 anti‐HSP70, Zombie UV Fixable Viability Kit, Alexa Fluor 647@anti‐mouse CD25, APC anti‐mouse CD3, FITC anti‐mouse CD8, PerCP/Cy5.5 anti‐mouse CD4, PE anti‐mouse CD11c, APC anti‐mouse CD80, PE/Cy7 anti‐mouse CD86 and PE anti‐mouse FOXP3 were purchased from Biolegend. Mouse ELISA kits for TNF‐α, IL‐6 and IFN‐γ were obtained from Immunoway.

### Preparation of DMC@P‐Cs

The DMC@P‐Cs were prepared via the solvent injection method. Briefly, CFL, DSPE‐PEG2000, DOPC, and pyropheophorbide‐lipid with molar ratios of 30%:5%:15%:50% (Total mass:1 mg) was first completely dissolved in ethanol and injected into PBS solution containing different amounts of DMC (1 mL) and water bath sonicated for 5 min, then probe‐sonicated with 10% output for 3 min was further performed in an ice‐water bath to obtain a more homogenized solution. Finally, the sample was incubated for 24 h in a 37 °C isothermal shaker to benefit the formation of silicate network on the cerasomes’ surface, followed by dialysis (MWCO: 8000−14 000 Da) to remove the organic solvent, and unencapsulated DMC was further removed by exclusion chromatography with Sephadex G‐50. Cerasomes with only sonosensitizer (P‐Cs) were prepared as a control. Lipoomes containing the sonosensitizer and DMC (DMC@P‐Ls) were prepared using the same procedure except using DSPC instead of CFL. In addition, the study had also used DSPC to replace PL for preparing non‐acoustic sensitizer cerasomes DMC@Cs.

### Characterizations

The morphology analysis of DMC@P‐Cs was carried out with a TEM (JEM‐1400, Japan). The hydrodynamic diameter and zeta potential of P‐Cs, DMC@P‐Ls, and DMC@P‐Cs were measured by a DLS analyzer (Zeta Size/NanoZS90, Netherlands). The fluorescence and absorbance spectra were measured on fluorescence (FL970) and absorption (UV2300II) spectrometers, respectively. Fourier transform infrared (FT‐IR) spectra were tested by an FT‐IR spectrophotometer (Thermo Nicolet iS5, USA).

### Measurement of ROS Generation

To evaluate the SDT performance of DMC@P‐Cs, a fluorescent probe of SOSG was used to evaluate the generated yield of singlet oxygen activated by US. 3 mL of DMC@P‐Cs suspension mixed with SOSG solution (6 µL, 5 mm) was exposed to ultrasonic irradiation for different time, duty cycle, power and concentrations, followed by fluorescence detection of SOSG at 510−600 nm.

### US‐Mediated Release of DMC

The release behaviors of DMC from DMC@P‐Cs and DMC@P‐Ls with or without US irradiation were investigated by the dialysis method, and free DMC was used as a control. The effectiveness of US triggering DMC release was confirmed by measuring the amount of released drug by absorption spectrophotometer. Different drug carriers containing 1 mg DMC were dispersed in PBS at 37 °C, followed by treatment with or without US irradiation at 0, 1, 2 h (US parameters: 1.0 MHz, 1.0 W cm^−2^, 20% duty cycle, 5 min). At various time‐points during sonication, the solution was sampled. The DMC release was calculated as a percentage of the total DMC in the drug carrier. Next, the influence of different ultrasonic parameters on DMC release was investigated using the same procedure.

### Stability Investigation of DMC@P‐Cs

Firstly, it was examined whether DMC@P‐Cs can maintain a stable state without aggregation and precipitation under physiological conditions. An appropriate amount of DMC@P‐Cs was taken and dispersed in PBS, Saline, FBS, RPMI‐1640 medium with 10% FBS and DMEM medium with 10% FBS, respectively. Photos were taken to observe the dispersion state in various solutions. Secondly, the morphological stability of cerasomes was investigated by Triton X‐100 solubilization experiment. Taking DMC@P‐Ls as the control group, Triton X‐100 with different concentrations was added into DMC@P‐Cs and DMC@P‐Ls nanoparticle solutions, and then the particle size changes were measured. All samples were tested for at least three times. In order to observe whether DMC@P‐Cs can maintain the stability of particle size in various media simulating physiological conditions, it was dispersed in the above four physiological media, and its particle size changes were monitored for seven consecutive days. DMC@P‐Ls was used as the control.

### Cell Culture Studies

Human umbilical vein endothelial cells (HUVECs) and CT26 murine colon carcinoma cells in a humidified atmosphere containing 5% CO_2_ were incubated in the RPMI‐1640 medium supplemented with 10% volume of fetal bovine serum (FBS) and 1% volume of a penicillin−streptomycin solution (PS) at 37 °C.

### Cellular Uptake In Vitro

Confocal laser scanning microscopy (CLSM, Nikon, Japan) and flow cytometry (Beckman CoulterCalibur2, USA) were used to qualitatively and quantitatively analyze the cellular uptake behavior of DMC@P‐Cs by CT26 cells. Typically, CT26 cells with a density of 1 × 10^5^ cells/dish were seeded in confocal dishes overnight, followed by the removal of culture media and replacement with fresh media containing PBS, PL, P‐Cs, and DMC@P‐Cs (PL concentration, 20 µg mL^−1^). After different time of incubation, the culture media were discarded and PBS washing was performed for several times, followed by lysosomes staining with lyso‐tracker Green. Then, 4% paraformaldehyde was added to fix the cells, which were further stained with DAPI for 3 min. Finally, the cells were imaged by CLSM using different bandpass filters for PL (*λex* = 405 nm; *λem* = 650−720 nm), lyso‐tracker Green (*λex* = 504 nm; *λem* = 511 nm) and DAPI (*λex* = 405 nm; *λem* = 410−490 nm), respectively. For flow cytometry, cells with a density of 1 × 10^6^ cells/well were seeded in the six‐well plates and treated with a similar procedure as mentioned above, the cells was washed with PBS and collected by centrifugation before flow cytometry analysis.

### Biocompatibility of P‐Cs

The biocompatibility of the P‐Cs was evaluated by the MTT assay and the LIVE/DEAD viability/cytotoxicity kit. For the MTT assay, HUVECs with density of 1 × 10^4^ cells/well were incubated in a 96‐well plate for 12 h, and then the medium was replaced with 200 µL of fresh medium containing different concentrations of P‐Cs and cultured for another 24 h. After discarding the medium and washing with PBS, the medium containing MTT (0.5 mg mL^−1^) was added and cultured for 4 h. Next, 200 µL of DMSO was added and placed on the shaker for 15 min. The absorbance at 490 and 630 nm were measured using a SPARK 10 M microplate reader, and cell viability was calculated according to the following equation (Equation 1).

(1) $$\mathrm{Cell}\;\mathrm{Viability}\;\;\left(\%\right)=\frac{OD_{490nm}\;sample-OD_{630nm}\;sample}{OD_{490nm}Blank-OD_{630nm}Blank}\;\times 100\%$$

For LIVE/DEAD cells staining assay, HUVECs in a 24‐well plate were incubated for 12 h. Then, the cells were exposed to P‐Cs at the same concentration as used for the MTT assay and incubated for 24 h. Finally, a LIVE/DEAD viability/cytotoxicity kit was added and observed with a fluorescence microscope.

### Hemolysis Assay

Fresh murine blood samples were harvested from BALB/c mice and centrifuged at 1000 g for 10 min to remove the supernatant and washed at least three times with PBS buffer. The red blood cells (RBCs) were resuspended in PBS buffer. RBCs suspension (100 µL) mixed with ddH_2_O (900 µL) was used as the positive control. RBCs suspension (100 µL) mixed with PBS buffer (900 µL) was used as the negative control. For DMC@P‐Cs groups, RBCs suspension (100 µL) was mixed with DMC@P‐Cs solutions (900 µL) at different final concentrations from 50 to 300 µg mL^−1^ and incubated for 8 h. Then all samples were centrifuged to precipitate RBCs (1000 g, 10 min). Then, photographs of all samples were captured, and the absorbance of supernatants at 570 nm were measured using a SPARK 10M  microplate reader. The hemolysis percentage was calculated according to the formula (Equation 2):

(2) $$Hemolysis\;\;\left(\%\right)=\frac{OD_{570nm}\;sample-OD_{570nm}\;ddPBS}{OD_{570nm}ddH2O-OD_{570nm}ddPBS}\;\times 100\%\;$$

where OD_570nm_ddH_2_O, OD_570nm_PBS and OD_570nm_ sample represented the absorbance of positive control, negative control and samples, respectively.

### Cytotoxicity Evaluation In Vitro

For cell viability experiment, CT26 cells at a density of 4000 cells/well were seeded in 96‐well plates and incubated overnight, and P‐Cs NPs or DMC@P‐Cs solutions (100 µL, PL concentrations: 0 to 100 µg mL^−1^) were added into wells. The cells without nanoparticle treatments were used as a control. The cells were then irradiated with US (1.0 MHz, 20% duty cycle, and 1.0 W cm^−2^) at 8 h post‐incubation for 4 min, and continuously incubated for another 24 h. After replaced with fresh culture media, MTT (100 µL, 0.5 mg mL^−1^ in RPMI‐1640) was added. 4 h later, the absorbance of MTT at 490 nm and 630 nm was measured using a SPARK 10M microplate reader to evaluate the cell viability.

### Detection of Intracellular ROS

CT26 cells in 24‐well cell culture dishes were seeded at a density of 8 × 10^4^ cells/dish for 24 h, followed by treatment with P‐Cs or DMC@P‐Cs for 12 h (PL concentration 20 µg mL^−1^). Then, each well was added with DCFH‐DA probe and further incubated for 1 h. The cells were irradiated with US for 4 min (1.0 MHz, 1.0 W cm^−2^, 20% duty cycle), followed by three times of washing with PBS before observing with a fluorescence microscope.

### Immunogenic Cell Death Induction

CT26 cells in a 6‐well culture plate (3 × 10^5^ cells/well) were incubated at 37 °C overnight to reach adherence. After diversified treatments (similar to those in MTT assay), paraformaldehyde (4%) was added to fix the tumor cells for 4 h, which was later permeated with Triton X‐100 (0.2%) for 5 min and blocked with BSA (1%) for 1 h at room temperature. Afterward, the treated cells were incubated with the primary antibodies of Alexa Fluor@594 anti‐HMGB1 (1.2 µg mL^−1^), Alexa Fluor@647 anti‐CRT (1.9 µg mL^−1^) or Alexa Fluor@488 anti‐HSP70 (1.5 µg mL^−1^) at 4 °C for 12 h. After 20 min of DAPI staining (1 µg mL^−1^), related intracellular fluorescence was measured through individual detection channels from CLSM.

### PP2A Phosphase Assay Measurement

CD4^+^ T cells were first obtained from splenocytes using the MojoSort Mouse CD4 T Cell Isolation Kit. Cells were activated using immobilized anti‐CD3 and CD28. For PP2A phosphatase assay, CD4^+^ T cells were first incubated with P‐Cs, DMC, DMC@P‐Cs (PL concentration: 12.5 µg mL^−1^, DMC concentration: 4 µg mL^−1^) for 8 h. For groups requiring, US irradiation was performed prior to incubation with cells for drug release. After collection, centrifugation, the cells were washed with PBS for three times, followed by lysing with 30 min of RIPA lysis buffer on ice, and the lysates were centrifuged (12 000 rpm, 20 min), and the supernatants containing the total cellular protein were then collected, and analyzed with Pierce BCA Protein Assay Kit for protein quantification. Then the lysate was used for PP2A phosphatase activity detection by Ser/Thr Phosphatase Assay Kit.

### Inhibition of Tregs Formation by DMC@P‐Cs

CD4^+^ T cells were incubated with TGF‐β with or without P‐Cs, DMC, DMC@P‐Cs (PL concentration:12.5 µg mL^−1^, DMC concentration: 4 µg mL^−1^) for 3 days, followed by collection, centrifugation and washing with PBS for several times, then blockage with anti‐mouse CD16/32 at 4 °C for 10 min, and fixation with Fixation Buffer for 20 min in the dark at room temperature. Intracellular Staining Perm Wash Buffer was used to resuspended the cells and PE anti‐mouse FOXP3 was added and incubated for 1 h. After three times of washing with PBS, the cells were examined with Fortessa X20 (BD Biosciences).

### Mouse Tumor Model Implantation

All animal experiments were carried out according to the Guidelines of the Peking University‐Institutional Animal Care and Use Committee (NTU‐IACUC) for Care and Use of Laboratory Animals, and Peking University Health Science Center supplied 6‐week‐old BALB/c mice for animal experiments. Designated ethical approval/approval number for animal experiments was LA2019206. PBS solution containing CT26 cancer cells were subcutaneously injected into each mouse on the right flank for tumor inoculation (1 × 10^6^ cells/mouse).

### In Vivo and Ex Vivo Fluorescence Imaging

Mice bearing CT26 tumor were used for fluorescence imaging (*n* = 3). DMC@P‐Cs and DMC@P‐Ls were intravenously injected into mice (PL injection dose = 4 mg kg^−1^). Fluorescence imaging of the mice was taken on an IVIS imaging system at different designed time‐points (*λ_ex_* = 675 nm, *λ_em_* = 720 nm). Fluorescence intensity quantification was performed using a Living Image software. At 48 h post‐injection, the tumors and major organs were dissociated from euthanized mice for ex‐vivo fluorescence imaging (*λ*_ex_ = 675 nm, *λ*_em_ = 720 nm).

### In Vivo Anti‐Tumor Efficacy and Histological Studies

When the mice bearing CT26 tumor grew to ≈100 mm^3^, six groups of mice were set up (*n* = 5), including PBS, P‐Cs, or DMC@P‐Cs (PL injected dose = 4 mg kg^−1^ mice) were then injected intravenously. After 24 h, ultrasound irradiation was performed on the primary tumors of each mouse (1 MHz, 50% duty, 1 W cm^−2^, 5 min). Mice tumor volumes and body weights were recorded every 3 days. Tumor volumes were calculated according to the following formula: tumor volume = (tumor length) × (tumor width)^2^/2. On day 19, the mice in all groups were euthanized and the tumors, tumor draining lymph nodes (TDLNs), and major organs were taken and fixed with 4% paraformaldehyde, followed by embedding and section. H&E staining, Tunel staining, Ki67 staining and Caspase‐3 staining were then carried out according to standard protocols, and observed with a digital microscope (Leica).

### In Vivo Evaluation of T Cell Population

At the end of the treatments, the primary tumors of CT26 tumor‐bearing mice were harvested to prepare single cell suspension. Briefly, small pieces of tumor tissues were first prepared and then digested at 37 °C for 4 h with a solution containing 100 µg mL^−1^ type IV collagenase, 1 mg mL^−1^ type I collagenase, and 100 µg mL^−1^ DNase I, followed by filtering through 70 µm cell strainer. The obtained single cell suspensions were mixed with anti‐mouse CD16/32 for blockage, followed by Zombie Yellow staining for discriminating live cells from dead cells. Then, anti‐CD3, anti‐CD4, anti‐CD8a were used to stain the cells at 4 °C for 30 min, which were analyzed by flow cytometry for CD4 and CD8 T cell populations. For Tregs analysis, anti‐mouse CD16/32 was mixed with single cell suspensions for blockage, followed by Zombie Yellow staining for discriminating live cells from dead cells. Then, anti‐CD3, anti‐CD4, anti‐CD25 were used to stain the cells at 4 °C for 30 min, and later fixed in the dark at room temperature in a 0.5 mL/tube Fixation Buffer for 20 min. Thereafter, Intracellular Staining Perm Wash Buffer was used to resuspend the cells, in which were incubated with PE anti‐mouse FOXP3 for 1 h, followed by Intracellular Staining Perm Wash Buffer washing for three times and analyzed with Fortessa X20 (BD Biosciences).

### DC Maturation Evaluation In Vivo

Mice bearing CT26 tumor with volume of ≈100 mm^3^ were divided into six groups (*n* = 3). PBS, P‐Cs, or DMC@P‐Cs (PL injected dose: 4 mg/kg mice) were then intravenously injected in to the mice. 24 h later, the primary tumors of each mouse were irradiated with ultrasound (1 MHz, 50%duty, 1 W cm^−2^, 5 min). On day 7, mice were euthanized to collect the tumor draining lymph nodes for preparing single cell suspension. The cells were first blocked with anti‐mouse CD16/32, followed by Zombie Yellow staining for discriminating live cells from dead cells. Then, anti‐CD11c, anti‐CD80, anti‐CD86 were used to stained the cells at 4 °C for 30 min, which were analyzed by flow cytometry after fixed by 4% paraformaldehyde for the maturation of DCs. As a blank control, spleen was also collected and cut into small pieces, followed by filtering through 70 µm cell strainer, and red blood cells in the splenocytes were removed by ACK lysis. Then they were incubated respectively with CD11c, CD80, CD86 at room temperature for 30 min as controls.

### Serum Cytokine Levels Measurement

On day 16, blood samples were collected from different groups of mice (*n* = 5), related levels of factors including TNF‐α, IL‐6, and IFN‐γ in serum were then detected with corresponding ELISA kit according to the manufacture protocols.

### Biosafety Measurement In Vivo

DMC@P‐Cs were intravenously injected into normal Balb/c mice (≈20 g) (PL concentration: 4 mg kg^−1^). On days 0, 1, 2, 7, and 14 after‐injection, blood samples were taken and the key indices of red blood cells (RBCs), platelets (PLTs), white blood cells (WBCs), mean corpuscular volume (MCV), hemoglobin (HGB), hematocrit (HCT), Platelet distribution width (PDW) and mean platelet volume (MPV), were measured via an automatic hematology analyzer according to the manufacturer's protocol.

### Statistical Analysis

The experimental results were expressed as mean ± SD. Statistical significance was calculated via one‐way ANOVA with a Tukey posthoc test. The differences were statistically significant for *p*‐value < 0.05 (^*^ *p* < 0.05, ^**^ *p* < 0.01, ^***^ *p* < 0.001, ^****^ *p* < 0.0001).

## Conflict of Interest

The authors declare no conflict of interest.

## Supporting information

Supporting Information

## Data Availability

The data that support the findings of this study are available from the corresponding author upon reasonable request.
