# Supplementary material for: Regulation of CTLs/Tregs via Highly Stable and Ultrasound‐Responsive Cerasomal Nano‐Modulators for Enhanced Colorectal Cancer Immunotherapy
Source: Adv Sci (Weinh). 2024 Mar 29;11(22):2400485. doi: 10.1002/advs.202400485 (PMC11165532; doi:10.1002/advs.202400485)
Supplement: Supplementary file 1 — Supporting Information [file ADVS-11-2400485-s001.pdf]

ADVANCED SCIENCE
Open Access

Supporting Information

for *Adv. Sci.*, DOI 10.1002/advs.202400485

Regulation of CTLs/Tregs via Highly Stable and Ultrasound-Responsive Cerasomal Nano-Modulators for Enhanced Colorectal Cancer Immunotherapy

Jinxia Zhang, Lihong Sun, Ling Jiang, Xinxin Xie, Yuan Wang, Ruiqi Wu, Qingshuang Tang, Suhui Sun, Shiwei Zhu, Xiaolong Liang and Ligang Cui**

Supporting Information

Regulation of CTLs/Tregs via Highly Stable and Ultrasound-Responsive Cerasomal Nano-Modulators for Enhanced Colorectal Cancer Immunotherapy

Jinxia Zhang[1,2], Lihong Sun[2], Ling Jiang[2], Xinxin Xie[2], Yuan Wang[2], Ruiqi Wu[2], Qingshuang Tang[2], Suhui Sun[2], Shiwei Zhu[2], Xiaolong Liang[1,2], Ligang Cui[1,2]**

[1]Institute of Medical Technology, Peking University Health Science Center, Beijing 100010, China

[2]Department of Ultrasound, Peking University Third Hospital, Beijing 100191 P. R. China

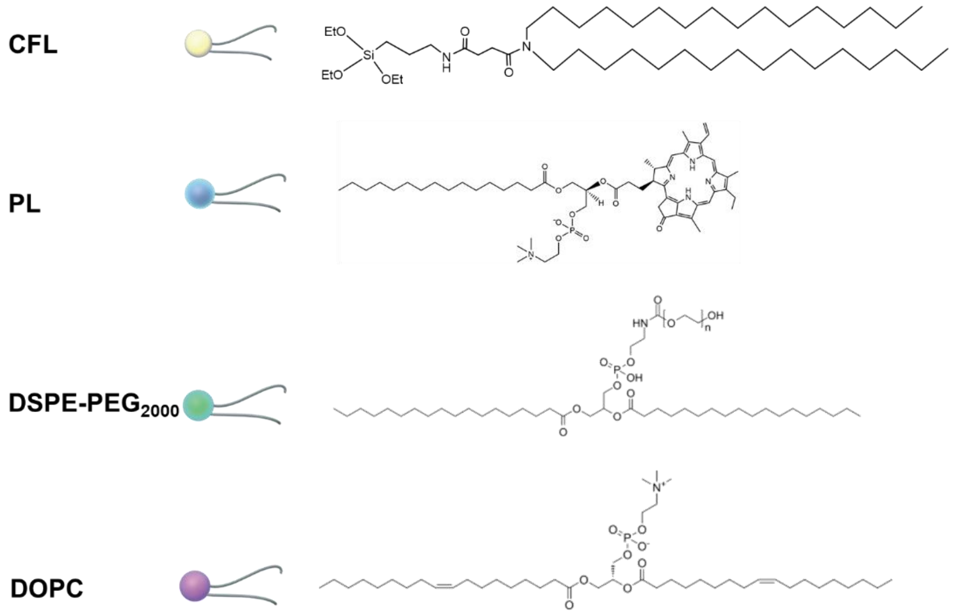

Figure S1. The chemical structures of various lipids.

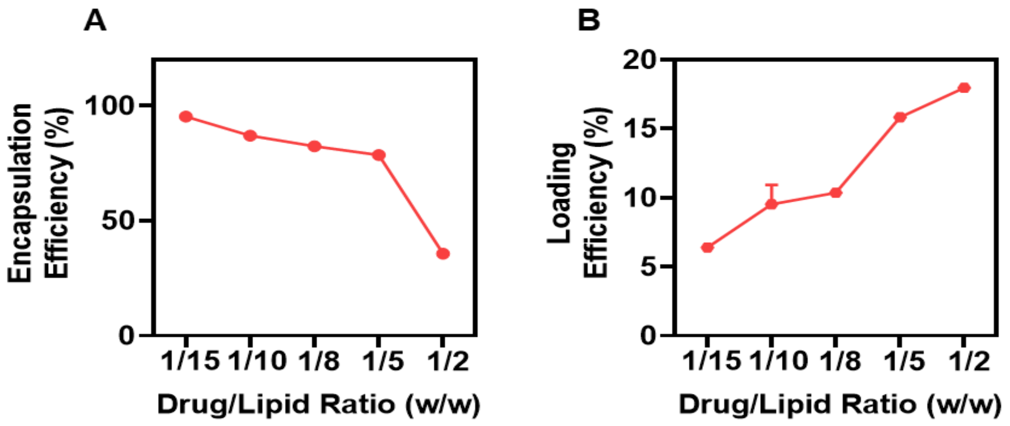

Figure S2. A. Encapsulation efficiency of DMC with different ratio of drug to lipid from 1/15 to 1/2; B. Loading efficiency of DMC with different ratio of drug to lipid from 1/15 to 1/2.

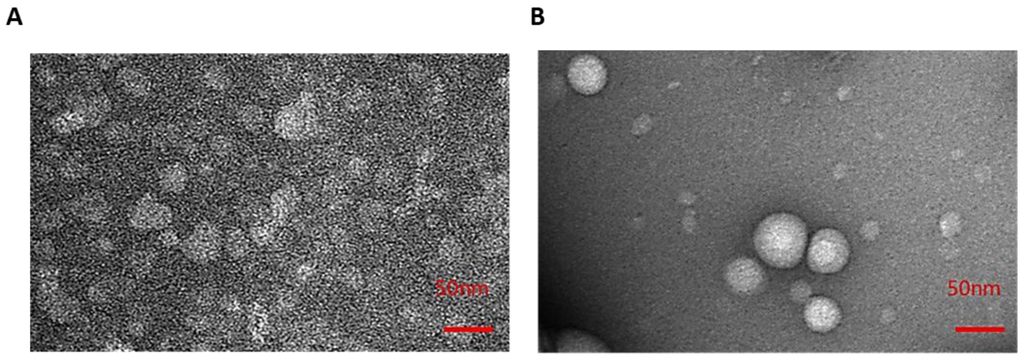

Figure S3. A. TEM images of P-Cs (Scale bar: 50 nm); B. TEM images of DMC@P-Ls (Scale bar: 50 nm).

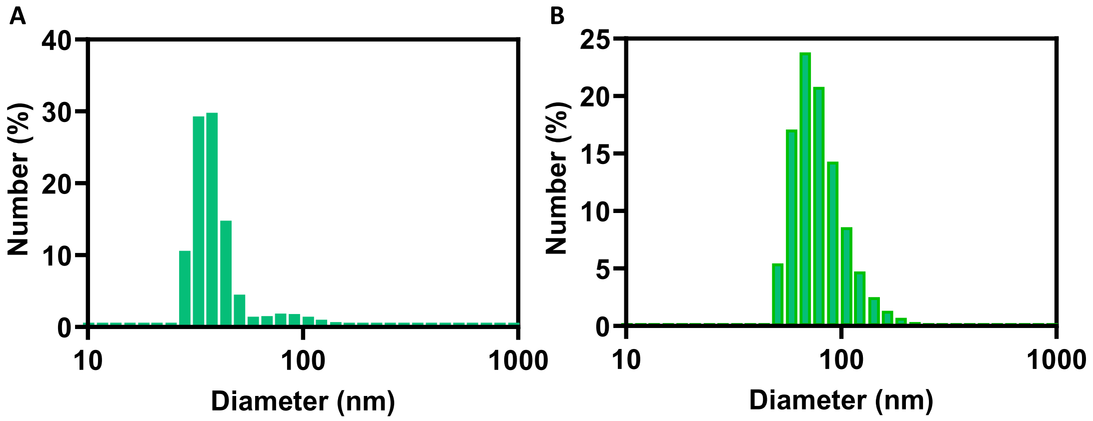

Figure S4. DLS measurements of (A) P-Cs and (B) DMC@P-Ls.

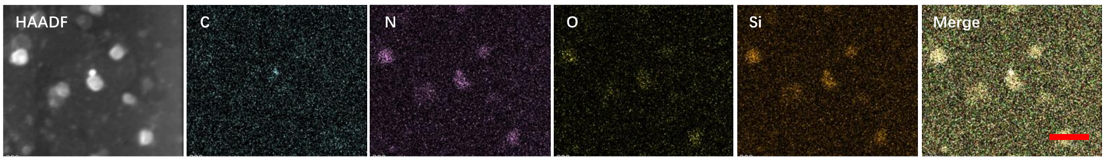

Figure S5. TEM element mapping images of DMC@P-Cs (Scale bar:100 nm).

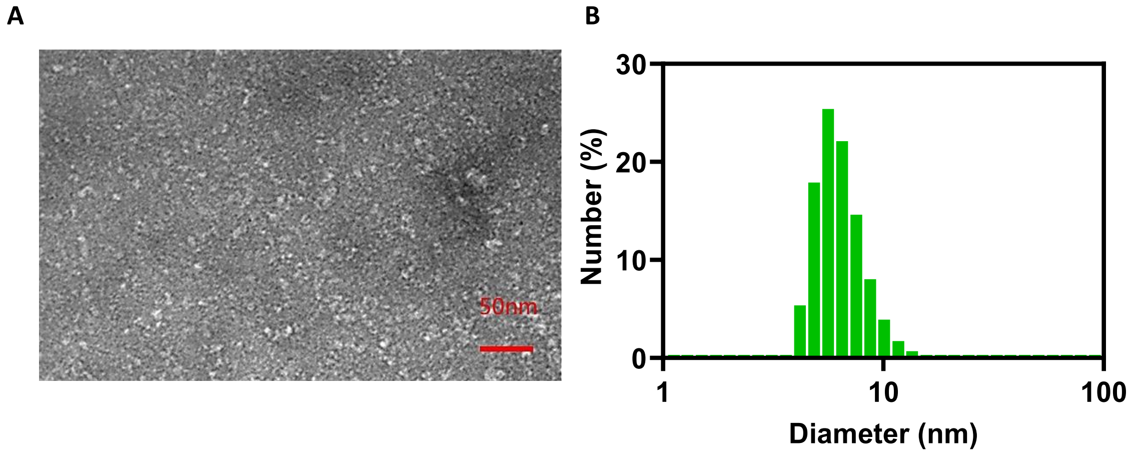

Figure S6. (A)TEM image (Scale bar:50 nm) and (B) DLS measurement of DMC@P-Cs with US irradiation.

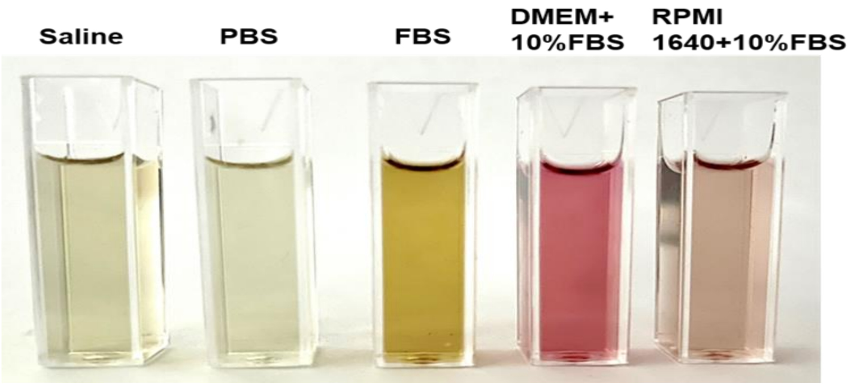

Figure S7. DMC@P-Cs dispersion in the following different media: Saline, PBS, FBS, DMEM+10% FBS, RPMI 1640+10% FBS.

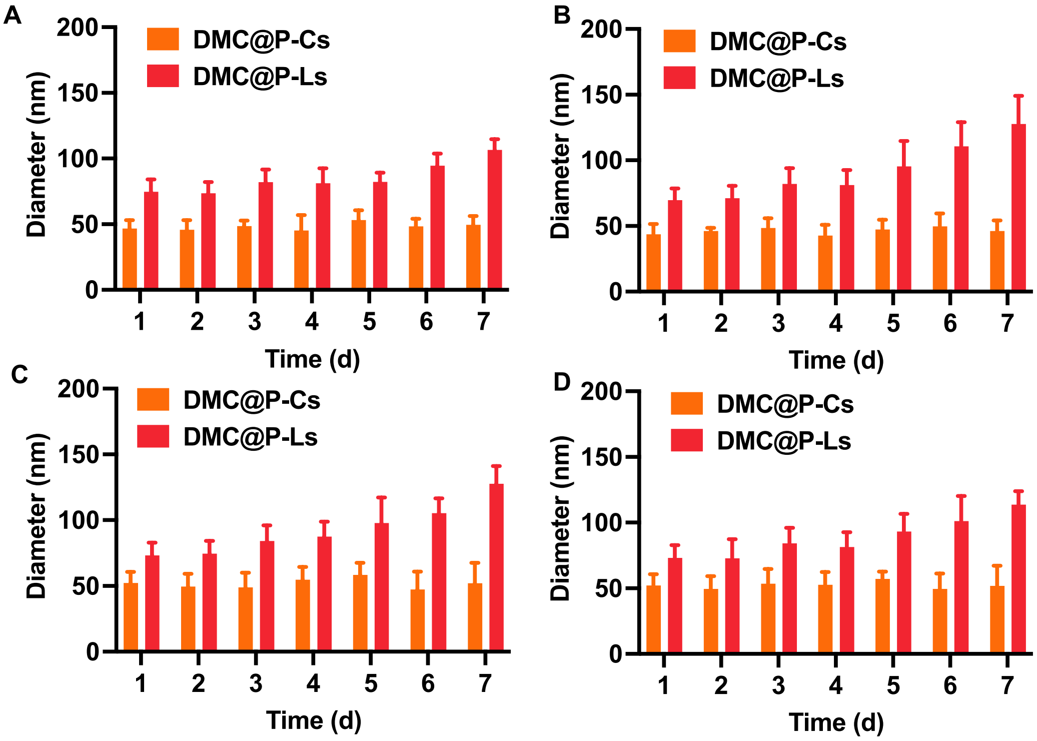

Figure S8. The time-dependence of diameter of DMC@P-Cs and DMC@P-Ls in PBS solutions (A), saline (B), DMEM+10%FBS (C) and RPMI 1640+10%FBS (D).

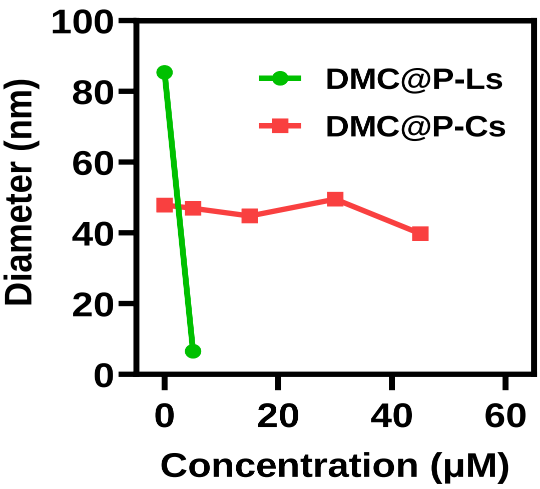

Figure S9. Effect of different concentrations of Triton-X100 on particle size of DMC@P-Cs and DMC@P-Ls.

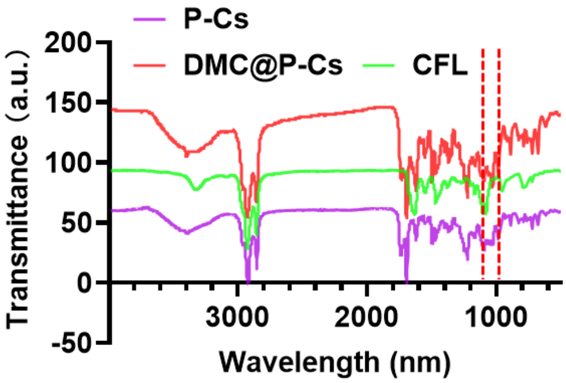

Figure S10. FTIR of CFL, P-Cs and DMC@P-Cs.

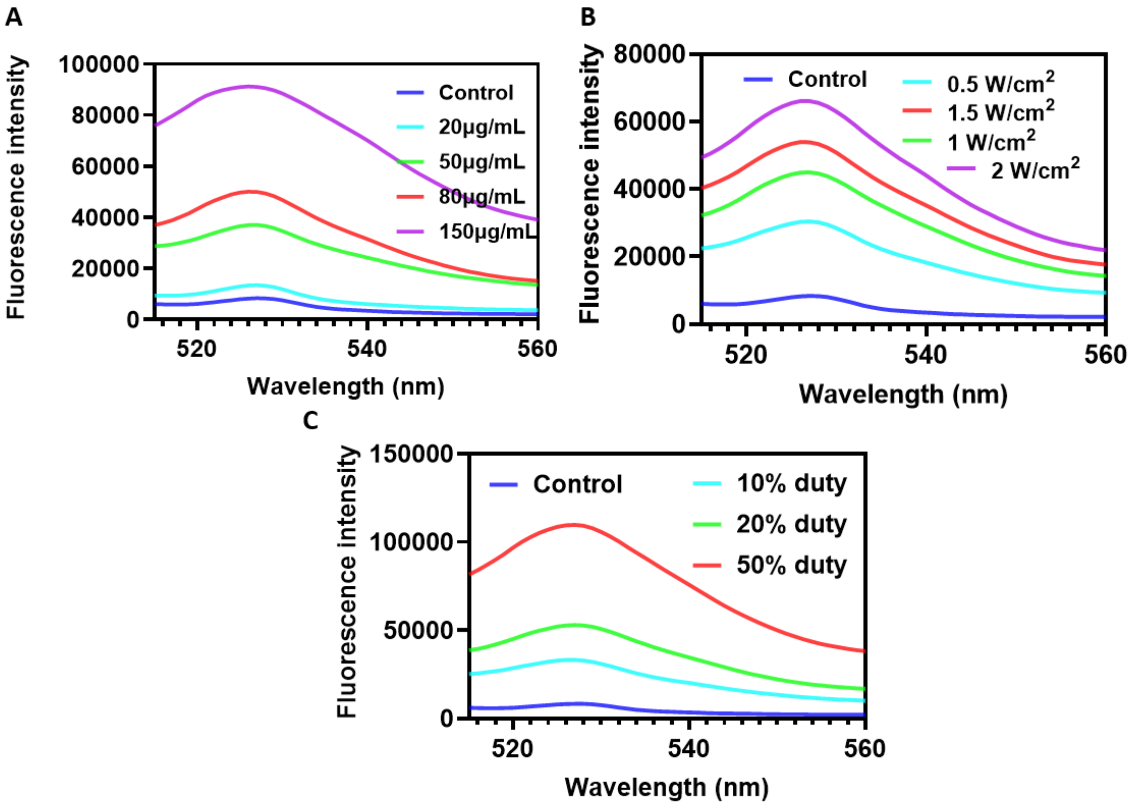

Figure S11. SOSG detected ROS produced by DMC@P-Cs under (A) different concentrations (represented by PL concentration); (B) different ultrasonic power density; (C) different ultrasonic duty cycle.

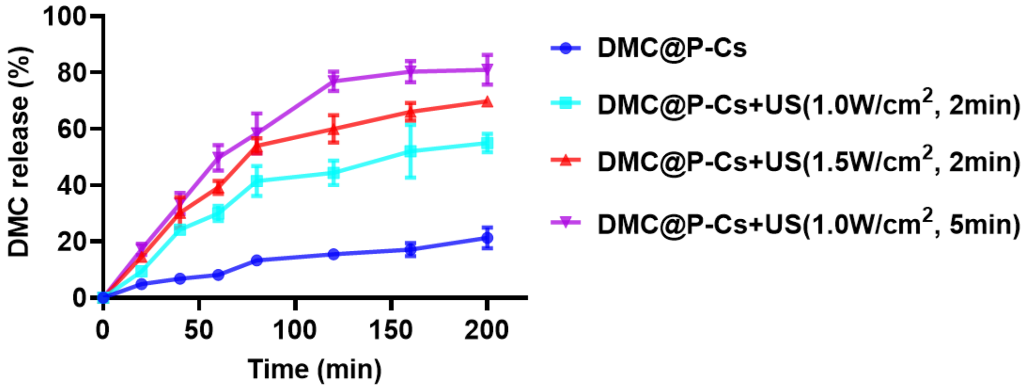

Figure S12. Effects of different ultrasonic parameters on DMC release behavior.

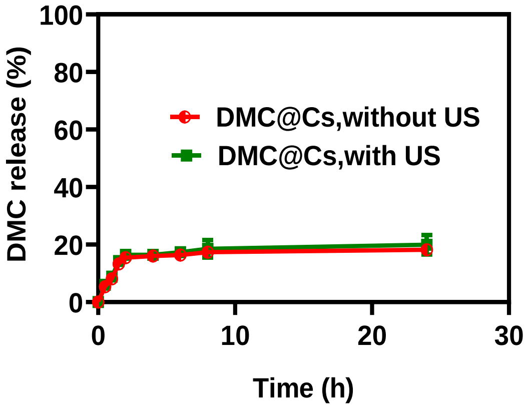

Figure S13. DMC release behavior from cerasomes without PL with US or without US irradiation.

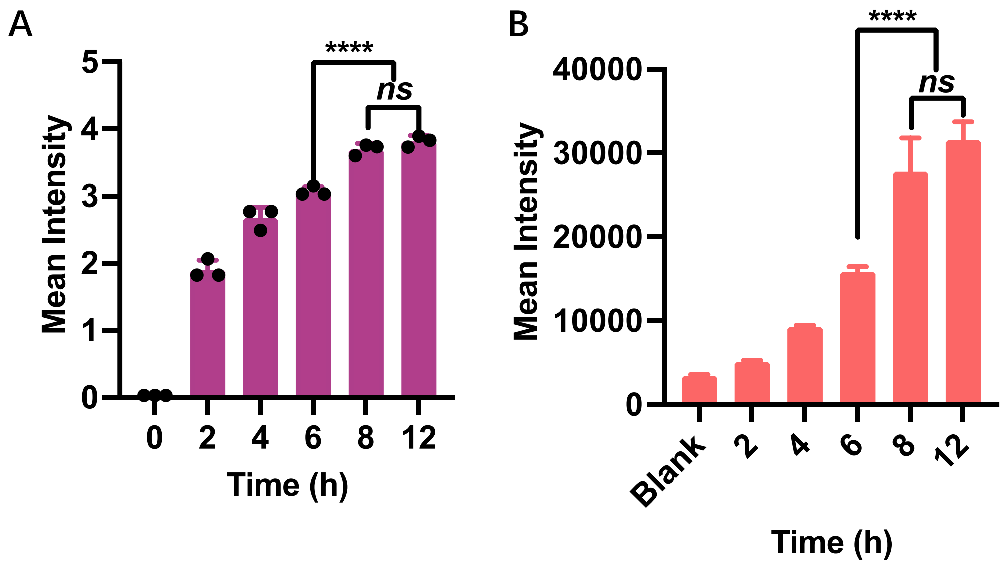

Figure S14. A. Semi-quantitative analysis of PL signal in Figure 3A. B. Quantitative analysis of PL signal in Figure 3B.

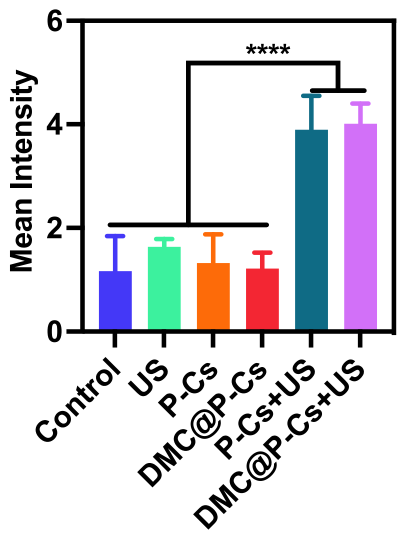

Figure S15. Semi-quantitative analysis of fluorescence intensity of ROS.

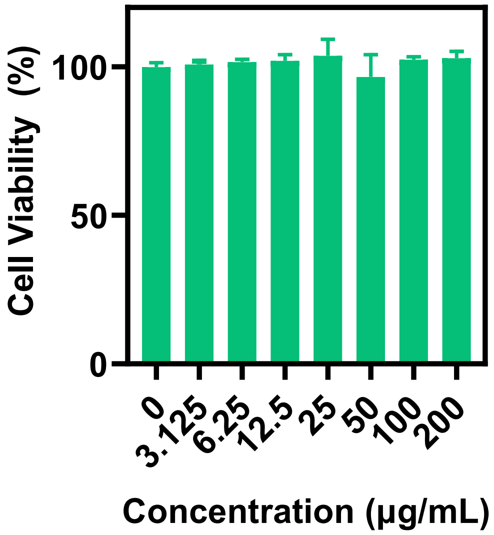

Figure S16. Viability of HUVECs incubated with different concentrations of P-Cs for 24 h.

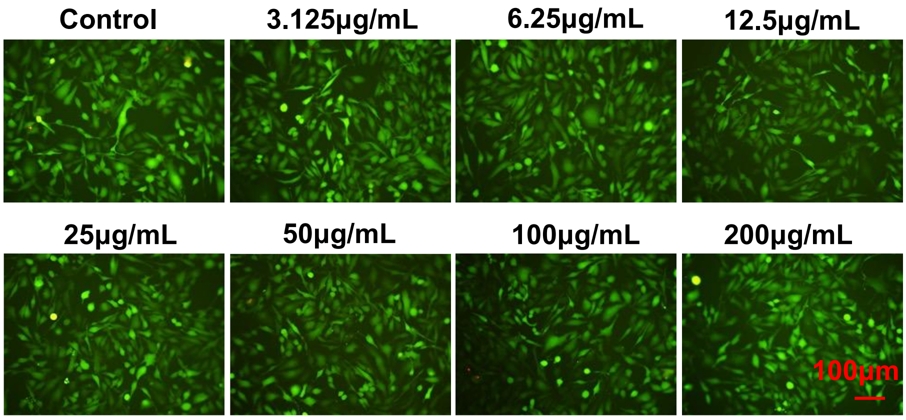

Figure S17. Fluorescence images of HUVEC cells stained with LIVE/DEAD activity /toxicity kit after treated with different concentrations of P-Cs. Scale bar:100μm.

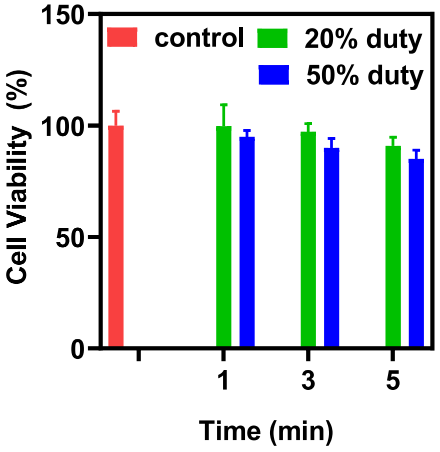

Figure S18. Cell viability of HUVEC cells was investigated after ultrasound treatment with different power and different time.

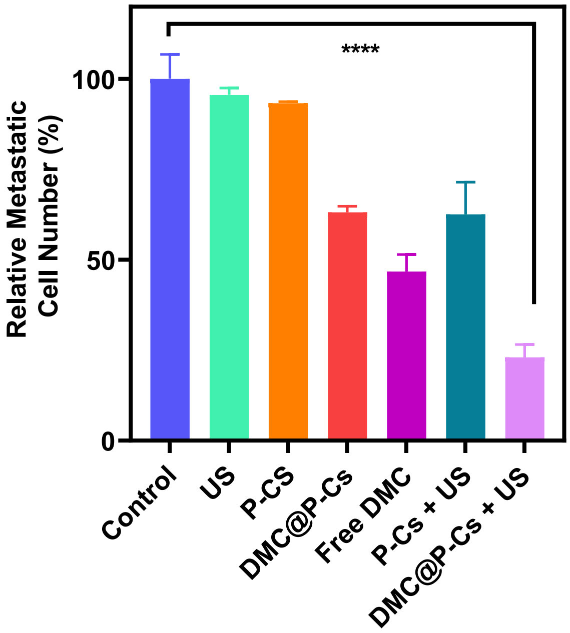

Figure S19. Quantitative analysis of relative number of cell invasion after different treatments.

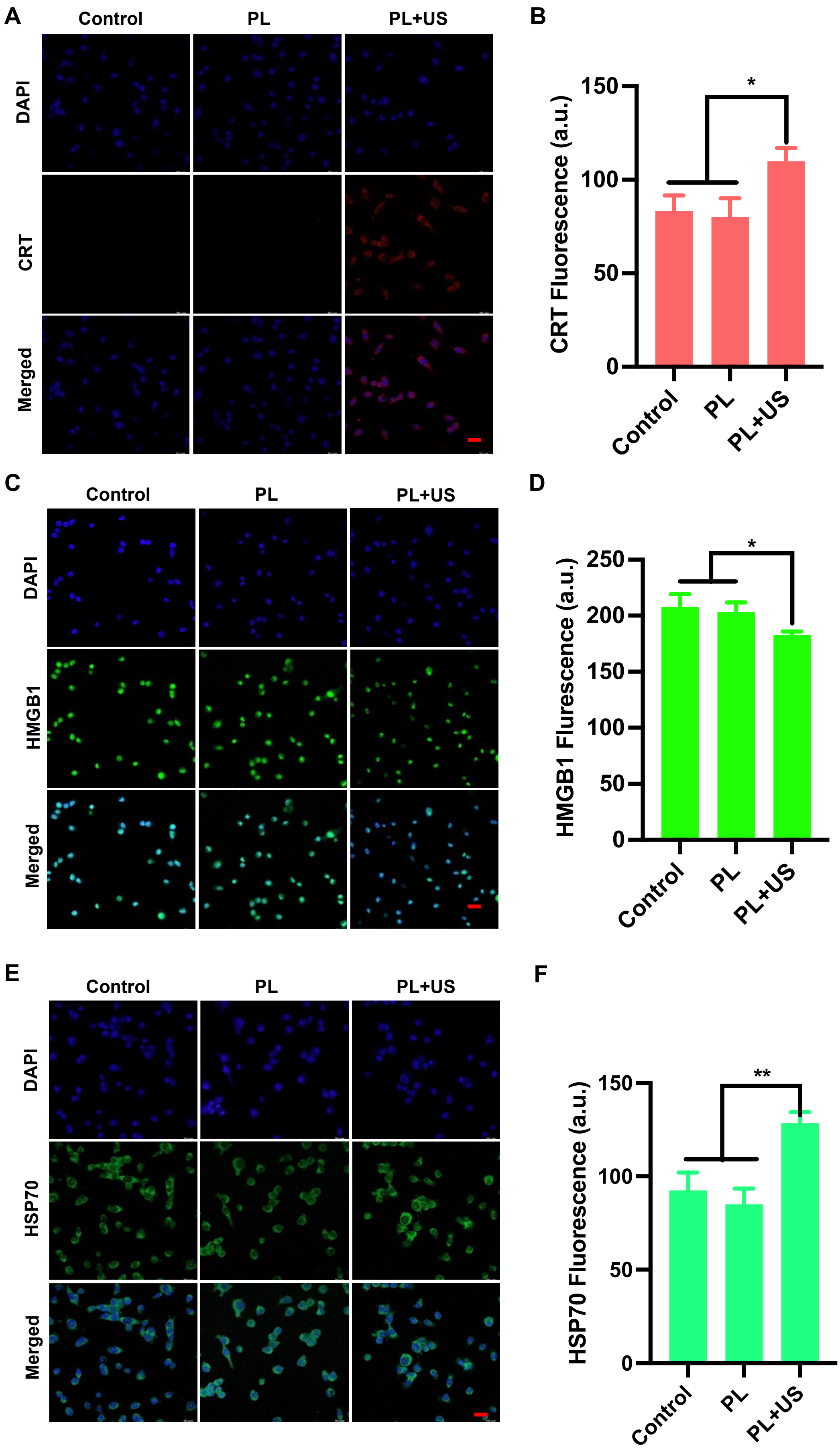
A
Control
PL
PL+US
DAPI
CRT
Merged
B
CRT Fluorescence (a.u.)
150
100
50
0
*
Control
PL
PL+US
C
Control
PL
PL+US
DAPI
HMGB1
Merged
D
HMGB1 Fluresecence (a.u.)
250
200
150
100
50
0
*
Control
PL
PL+US
E
Control
PL
PL+US
DAPI
HSP70
Merged
F
HSP70 Fluorescence (a.u.)
150
100
50
0
**
Control
PL
PL+US

Figure S20. SDT-Triggered ICD induced by PL+US. A. CRT exposed on CT26 cell surface after different treatments as observed by CLSM (Scale bar: 20 μm). B. Quantification of CRT signal intensity for different groups in Figure S20A. Data are presented as mean ± SD (n = 3). C. HMGB1 released from CT26 cells after different treatments as observed by CLSM (Scale bar: 20 μm). D. Quantification of HMGB1 signal intensity for different groups in Figure S20C. Data are presented as mean ± SD (n = 3). E. HSP70 exposed on CT26 cell surface after different treatments as observed by CLSM (Scale bar: 20 μm). F. Quantification of HSP70 signal intensity for different groups in Figure S20E. Data are presented as mean ± SD (n = 3).

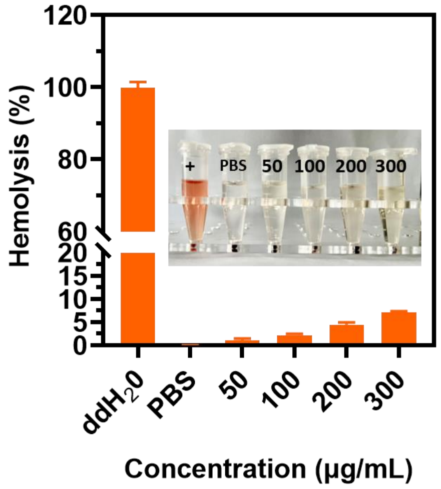

Figure S21. Hemocompatibility tests by incubating RBCs in the dispersion of DMC@P-Cs at various concentrations. DI water and PBS were used as positive (+) and negative (-) controls, respectively.

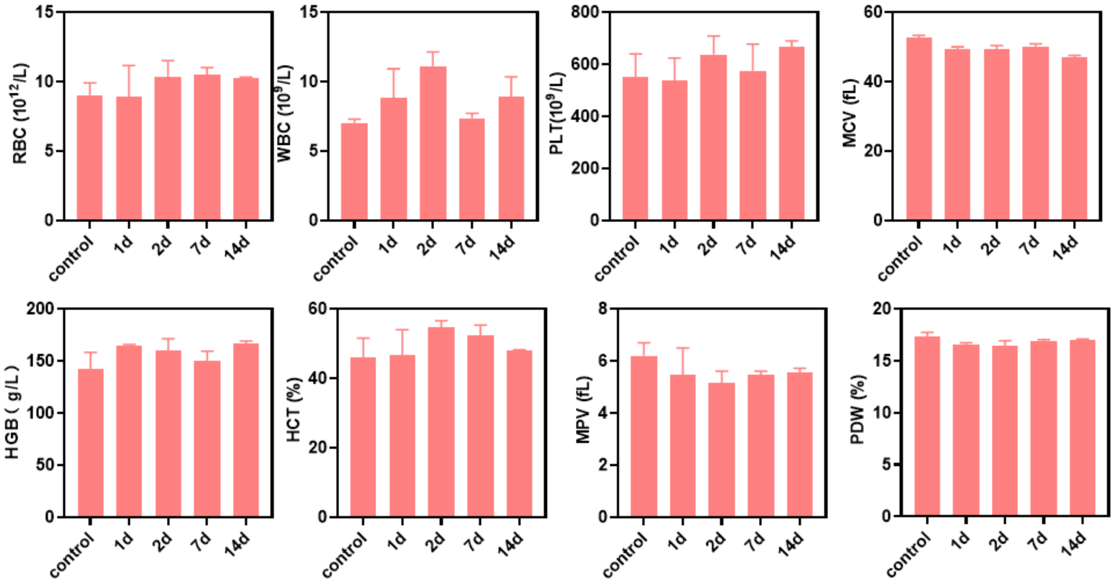

Figure S22. Primary indicators of blood routine tests of mice at days 1, 2, 7, and 14 after injection of DMC@P-Cs.

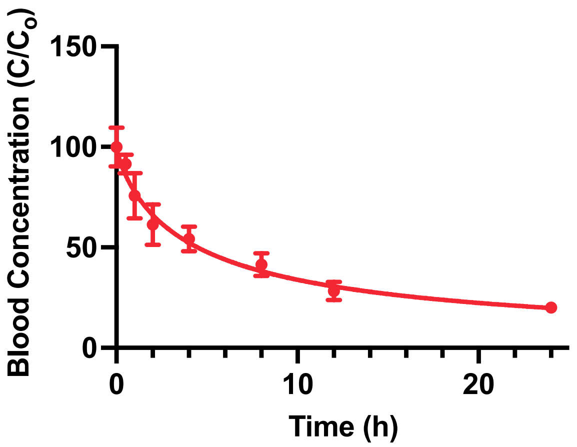

Figure S23. Pharmacokinetics of DMC over 24 h after intravenous injection of the DMC@P-Cs.

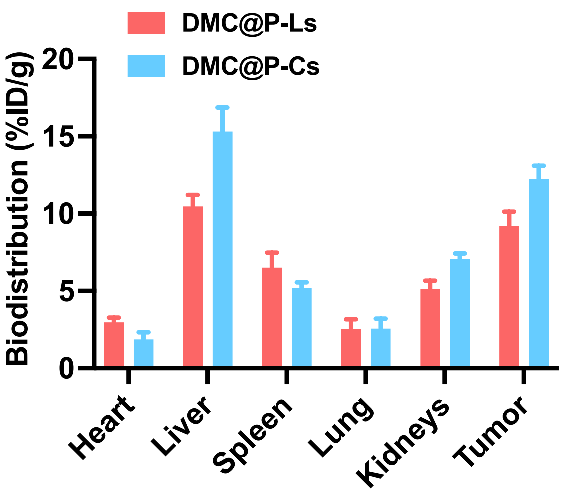

Figure S24. Biological distribution of tumor-bearing mice after different treatments.

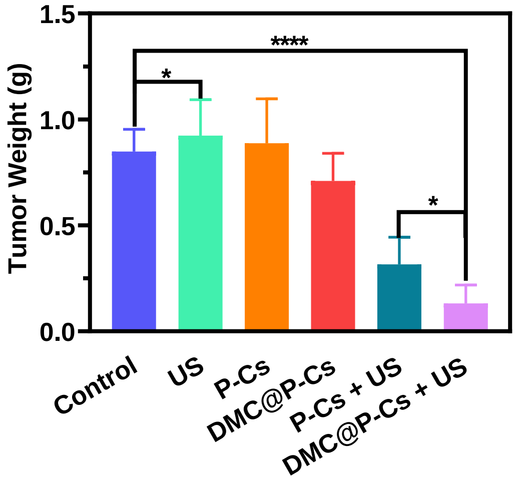

Figure S25. Tumor weight of different groups of CT26 tumor taken at day19.

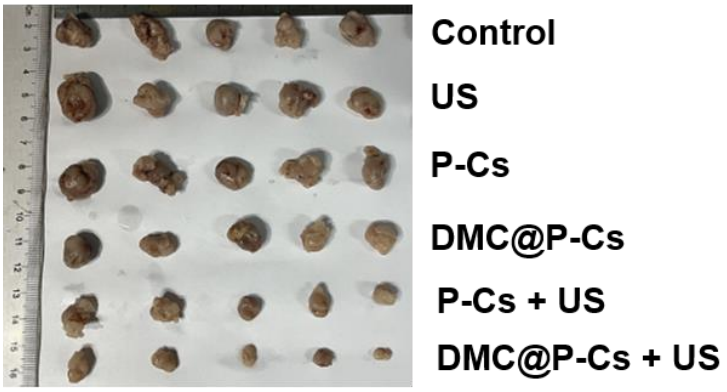

Figure S26. Photographs of CT26 tumors taken at day19.

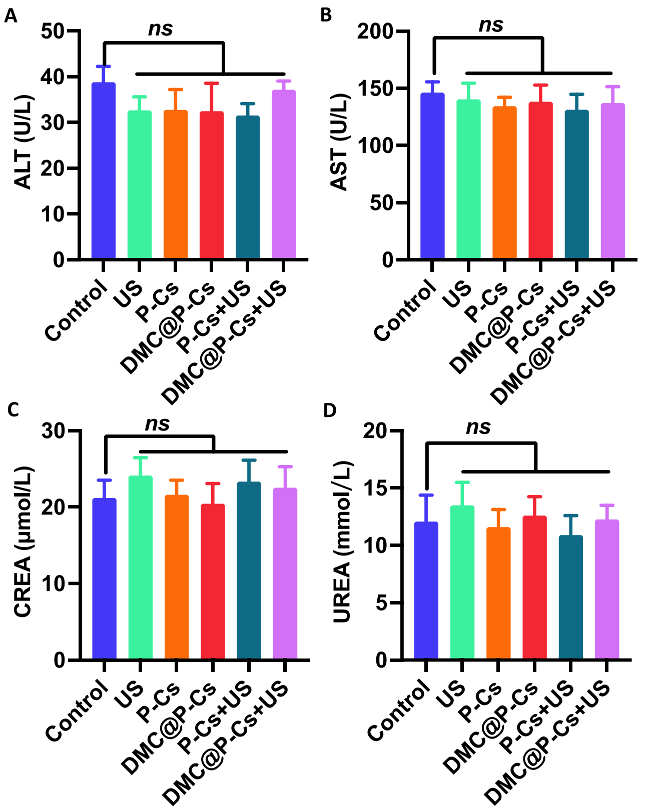

Figure S27. Parameters of blood biochemistry examination. A. ALT; B. AST; C. CREA; D. UREA.

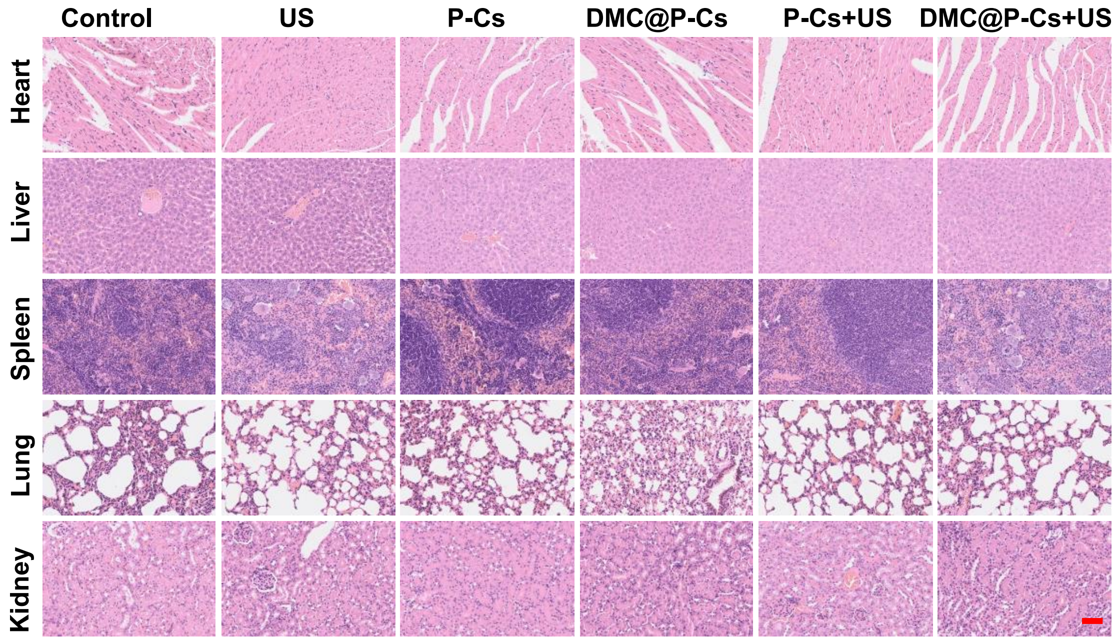

Figure S28. H&E staining of the major organ sections at day 19 after different treatments (Scale bar: 100 μm).

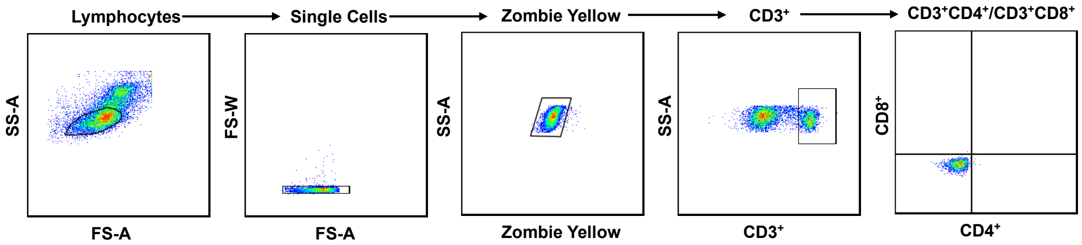

Figure S29. Representative gating strategy for flow cytometry assay of CTLs in tumors.
